# A novel inhibitor of human cytomegalovirus acts through a specific antiviral mechanism that involves the helicase-primase complex

**DOI:** 10.1128/aac.01977-25

**Published:** 2026-07-13

**Authors:** Arun Kapoor, Ayan K. Ghosh, Michael Forman, Alyza Skaist, Sarah Wheelan, Ravit Arav-Boger

**Affiliations:** 1 Department of Pediatrics, Division of Infectious Diseases, Medical College of Wisconsin 5506, Milwaukee, Wisconsin, USA; 2 Department of Pathology, Johns Hopkins University School of Medicine 1500, Baltimore, Maryland, USA; 3 Department of Molecular Biology and Genetics, Sidney Kimmel Comprehensive Cancer Center, Johns Hopkins University School of Medicine 1500, Baltimore, Maryland, USA; 4 Department of Genetic Medicine, Sidney Kimmel Comprehensive Cancer Center, Johns Hopkins University School of Medicine, Baltimore, Maryland, USA; 5 Department of Oncology, Sidney Kimmel Comprehensive Cancer Center, Johns Hopkins University School of Medicine, Baltimore, Maryland, USA; Chinese Academy of Medical Sciences & Peking Union Medical College, Beijing, China

**Keywords:** human cytomegalovirus, helicase-primase, primase-associated factor, drug target

## Abstract

Human cytomegalovirus (HCMV) remains the leading viral cause of congenital birth defects and a significant pathogen in transplant recipients. The most commonly used drugs inhibit the viral DNA polymerase, but result in severe side effects and the emergence of drug resistance. The terminase inhibitor (letermovir) and viral UL97 kinase inhibitor (maribavir) have improved the CMV armamentarium; however, both select for resistance, and have limited therapeutic indications. We recently identified MLS8091, a highly selective HCMV inhibitor. While its maximal time of HCMV inhibition overlaps with ganciclovir (GCV), it remains active against GCV-resistant strains, suggesting a distinct mechanism involving the CMV replisome. Using resistance selection protocols and next-generation sequencing of mutant viruses that escaped MLS8091 inhibition, we identified point mutations in the HCMV primase (UL70 D486H, D487Y) and the primase-associated factor (UL102 P165S, F275L) of the helicase-primase (H/P) complex. Marker transfer experiments confirmed that UL70 D486H, UL70 D487Y, and UL102 F275L each conferred resistance to MLS8091. UL102 P165S was identified together with UL70 D487Y; however, its independent contribution to resistance was not determined. Sequencing of 17 wild-type and GCV-resistant clinical isolates identified a UL102 polymorphism, A294G, that did not confer reduced susceptibility to MLS8091 upon marker transfer. Among the herpesviruses tested, only guinea pig CMV (GPCMV) was moderately inhibited by MLS8091. Sequence alignment revealed some similarity between GPCMV and HCMV UL70 486–487. In summary, we identified an inhibitor of HCMV replication that acts through a novel mechanism involving the H/P complex, opening new avenues in the development of HCMV-specific drugs.

## INTRODUCTION

Infection with human cytomegalovirus (HCMV), a member of the herpesvirus family, is common in humans. Seroprevalence rates increase with age, reaching 90% in individuals aged 80 years or older (1). HCMV establishes lifelong persistent infection, and patients typically remain asymptomatic. HCMV is the most common congenital infection worldwide (2–4). It is the leading infectious cause of hearing loss, intellectual disability, and central nervous system damage in children. The estimated annual societal cost of supporting children with congenital HCMV now approaches $4 billion (5). In immunocompromised hosts, HCMV causes significant morbidity and mortality (6, 7). More than 75% of solid organ transplant (SOT) recipients are newly infected or reactivate latent HCMV after transplantation. Human Immunodeficiency Virus (HIV)-infected patients who are HCMV-seropositive progress more rapidly to acquired immunodeficiency syndrome (AIDS) than those who are HCMV-seronegative (8, 9). HCMV may also enhance mother-to-child transmission of HIV (10). Despite significant advances in antiretroviral therapy, HCMV retinitis and organ disease are still reported in patients with AIDS (11). The most common anti-HCMV drugs target the viral DNA polymerase (12, 13). Their use is associated with toxicities to the bone marrow (ganciclovir [GCV]) and kidneys (foscarnet and cidofovir), as well as the emergence of resistant viruses (12, 14, 15). A Phase III clinical trial documented transient prevention of hearing loss in children treated with intravenous GCV, paving the way for treating those with congenital HCMV and central nervous system disease (16). A follow-up Phase III clinical trial of oral valganciclovir (the valyl ester prodrug of ganciclovir) in congenitally infected infants suggested a modest neurobehavioral benefit for 6 months compared to 6 weeks of therapy (17). Widespread use of a limited number of drugs leads to the development of drug-resistant strains (18, 19). Letermovir, a terminase inhibitor, is approved for HCMV prophylaxis after hematopoietic stem cell transplantation (20, 21), and resistance has already been reported (19, 22). Maribavir (MBV), an inhibitor of HCMV UL97 kinase, showed promising results (23, 24). In a Phase II clinical trial of resistant/refractory HCMV, a response rate of approximately 65% was observed across all MBV doses, defined as undetectable viral load within 6 weeks of treatment. There were no significant safety issues or bone marrow suppression; however, HCMV recurrences occurred in 35% of participants (25). In a phase III clinical trial, MBV was superior to conventional antiviral therapy (26). This led to its Food and Drug Administration (FDA) approval for adults and children (>12 years) with resistant/refractory HCMV disease. A recent subgroup analysis of SOT recipients demonstrated the greater effectiveness of MBV in clearing CMV DNAemia and fewer discontinuations due to treatment-emergent adverse events compared with investigator-assigned therapy (27).

The overall problems of toxicity, resistance, oral bioavailability, and high cost limit the effectiveness of approved anti-HCMV agents (28). There is a critical need for new drugs with distinct mechanisms of virus inhibition, high oral bioavailability, and low toxicity. We recently completed the largest reported high-throughput screen (HTS) of ~400,000 compounds for HCMV inhibitors and identified five structurally unique hits active at sub-low micromolar concentrations (29). We report here on compound MLS8091, which has a novel mechanism of action targeting the HCMV helicase-primase (H/P) complex.

## MATERIALS AND METHODS

### Compounds

**Fig 1:**
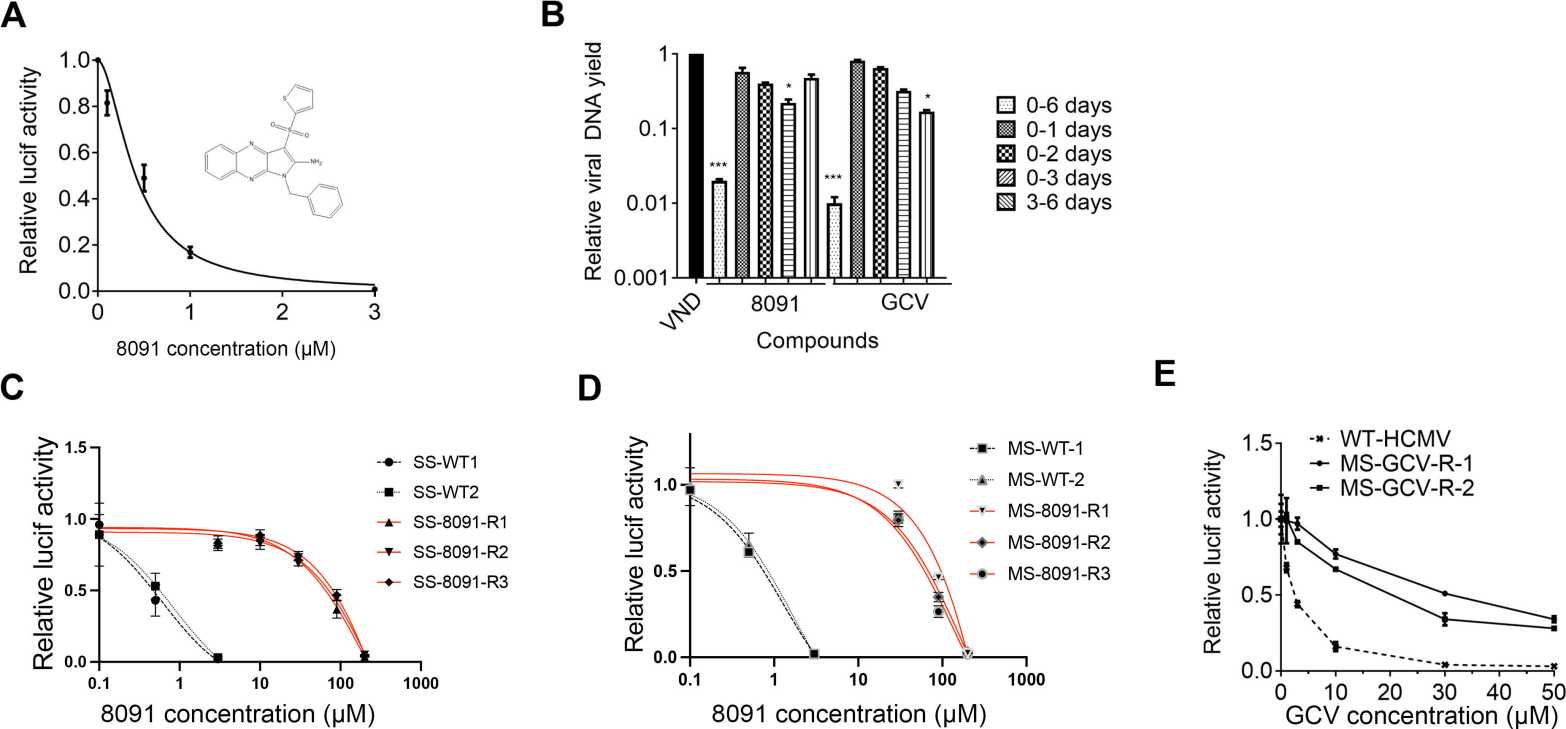
Anti-HCMV activity of MLS8091 against WT and drug-resistant mutants. (**A**) Human foreskin fibroblasts (HFFs) were infected with HCMV Towne (multiplicity of infection [MOI] of 1 PFU/cell) and treated with the indicated concentrations of MLS8091 (chemical structure). Luciferase activity was measured in cell lysates at 96 h post-infection (hpi). (**B**) HFFs were infected with HCMV Towne (MOI 1) and treated with either MLS8091 (3 µM) or GCV (5 µM) for the indicated time points. Viral DNA was isolated from cell supernatants at day 6 and quantified by US17 real-time PCR. Conditions included continuous compound exposure through day 6, compound removal at days 1, 2, or 3, or delayed compound addition at day 3 with removal at day 6. Statistical significance is compared with the virus-no-drug (VND) conditions. (**C**) HFFs were infected with pp28-luciferase wild-type (MOI 0.1) or MLS8091-resistant mutants generated by a single-step (SS) protocol and treated with the indicated concentrations of MLS8091. Luciferase activity was measured in cell lysates at 72 hpi. R1, 2, and 3 represent individual viral strains from SS selection. Each strain was sequenced after 10 drug-free passages following the initial SS selection and plaque purification. Data for R1, R2, and R3 refer to the stocks collected after 10 drug-free passages. (**D**) HFFs were infected with wild-type or MLS8091-resistant mutants generated by a multi-step (MS) protocol and treated with the indicated concentrations of MLS8091. Luciferase activity was measured in cell lysates at 72 hpi. R1, 2, and 3 represent individual viral strains from MS selection. Each strain was sequenced after 10 drug-free passages following the initial MS selection and plaque purification. Data for R1, R2, and R3 refer to the stocks collected after 10 drug-free passages. (**E**) HFFs were infected (MOI 0.1) with wild-type or GCV-resistant mutants generated by the MS protocol, and pp28 luciferase was measured at 72 hpi.

MLS8091(29) (Fig. 1) was purchased from Princeton Biomolecular Research (OSSK_752015) and dissolved in dimethyl sulfoxide (DMSO). Stock solutions of 10 and 50 mM were stored at −­80°C. Ganciclovir was purchased from Sigma-Aldrich (St. Louis, MO), and cidofovir from AK Scientific (Cat #G721). A 10 mM stock solution was prepared in deionized water (ddH2O) and stored at −80°C.

### Cells

Human Foreskin fibroblasts (HFFs), passages 12–16 (ATCC, CRL-2088), were grown in Dulbecco’s modified Eagle medium (DMEM) containing 10% fetal bovine serum (FBS) (Gibco, Carlsbad, CA) in a 5% CO2 incubator at 37°C.

### Viruses and antiviral assays

A recombinant pp28-luciferase Towne HCMV strain expressing luciferase under the control of the UL99 (pp28) late promoter was used (30). HCMV strain AD169 in bacterial artificial chromosome (BAC, pAD/Cre) was kindly provided by Dr. Rob Kalejta, University of Wisconsin–Madison. Wild-type and UL70 or UL102-resistant mutants were reconstituted from AD169-BAC. HCMV clinical isolates were obtained from the Johns Hopkins Microbiology laboratory without identifiers that can link to specific patients. Guinea pig CMV (GPCMV) was obtained from ATCC (VR-682).

### Luciferase activity

Cell lysates were collected at 72 h post-infection (hpi), and luciferase activity was measured using the Glomax-Multi + Detection System (Promega, Madison, WI) as previously described (30).

### Plaque reduction assays

HFFs were seeded into 12-well plates (1 × 10^5^ cells/well) and infected with wild-type or MLS8091-resistant HCMV (Towne or AD169) at approximately 100–200 plaques/well. After 90 min, the media were aspirated, and DMEM containing 0.5% carboxymethylcellulose (CMC), 4% fetal bovine serum (FBS), and the appropriate drugs were added to the triplicate wells. After incubation at 37°C for approximately 10 days, the overlay was removed, and plaques were stained with crystal violet and counted.

Guinea pig (GP) lung fibroblasts (ATCC CCL-158) were used for infection with GPCMV. For plaque assays, cells were seeded at 1.6 × 10^5^ cells per well in a 12-well plate and infected 24 h later with GPCMV at 100 PFU/well. Following a 90-min adsorption period, the medium was aspirated, and a fresh medium containing MLS8091 at the indicated µM concentrations, 0.5% carboxymethylcellulose, and 4% fetal bovine serum (FBS) was added to duplicate wells. After incubation at 37°C for 5 days, the overlay was removed, and the monolayer was stained with crystal violet. Plaques were counted microscopically under low power (40×). The effect of MLS8091 was calculated as the percent reduction in plaque number at each concentration relative to the number observed in its absence.

### DNA isolation and quantitative real-time PCR (qPCR)

Total DNA was isolated from non-infected and HCMV-infected HFFs using a Wizard SV Genomic DNA Isolation Kit (Promega, Madison, WI). To determine the viral load in the supernatant, total DNA was isolated using automated DNA extraction on the BioRobot M48 instrument (Qiagen, Valencia, CA). A US17 real-time PCR assay targeting a 151-bp region within the highly conserved US17 locus of the HCMV genome was used (31). The primers and probe for US17 are: forward- 5′ GCGTGCTTTTTAGCCTCTGCA-3′, reverse 5’- AAAAGTTTGTGCCCCAACGGTA-3′, and US17 probe FAM- 5′ TGATCGGGCGTTATCGCGTTCT-3′.

For GPCMV, CCL-158 cells were infected at an MOI of 0.1 and treated with MLS8091 or cidofovir (CDV, 5 µM) as a positive control. DNA was isolated at 3 days post-infection (DPI) with the SV Genomic DNA Isolation Kit (Promega, Madison, WI), and qPCR was performed for UL83 with the following primers: UL83 F6 (5′-CGACGACGACGATGACGAAAAC-3′) and UL83B11 (5′-TCCTCGGTCTCAACGAAGGGTC-3′) (32). This primer pair amplifies a 248-base pair region. GP GAPDH primers: F′GGGCAAGGTCATCCCAGAG, GAPDH R′TGGAAGAATGGCTGTCACTGT.

### SDS-polyacrylamide gel electrophoresis and immunoblot analysis

Cell lysates containing an equal amount of proteins were mixed with an equal volume of 2× sample buffer (125 mM Tris-HCl, pH 6.8, 4% SDS, 20% glycerol, and 5% β-mercaptoethanol) and boiled at 100°C for 10 min. Denatured proteins were resolved in Tris-glycine polyacrylamide gels (8%–10%) and transferred to polyvinylidene difluoride (PVDF) membranes (Bio-Rad Laboratories, Hercules, CA) by electroblotting. Membranes were incubated in blocking solution (5% wt/vol non-fat dry milk and 0.1% Tween-20 in PBS [PBST]) for 1 h, washed with PBST, and incubated with primary antibodies at 4°C overnight. Membranes were washed with PBST and incubated with horseradish peroxidase-conjugated secondary antibodies in PBST for 1 h at room temperature. Following washing with PBST, protein bands were visualized by chemiluminescence using SuperSignal West Dura and Pico reagents (Thermo Fisher Scientific, Rockford, IL). The following antibodies were used: mouse monoclonal anti-HCMV IE1 & IE2 (MAB810, Millipore, Billerica, MA), mouse monoclonal anti-HCMV UL83 (pp65, Vector Laboratories Inc., Burlingame, CA), mouse monoclonal anti-HCMV UL44, mouse monoclonal anti-HCMV UL84, and mouse anti-β-actin anti-mouse IgG (Santa Cruz Biotechnology, Santa Cruz, CA). Horseradish peroxidase (HRP)-conjugated anti-mouse IgG was from GE Healthcare (Waukesha, WI). Polyclonal antibodies to HCMV UL70 and UL102 were produced at Covance Inc. (Princeton, New Jersey). The following peptides were used for the generation of antibodies in SPF rabbits: UL70: AC-CHSNAKNVHISIKIRPPDAP-AMIDE and UL102: AC-CQHAAKRVASEGLRFFRLNA-OH.

### Plasmids

A pp71 expression plasmid was obtained from Dr. Rob Kalejta, University of Wisconsin–Madison. The pEPKan-S plasmid was obtained from Dr. Prashant Desai, Johns Hopkins University School of Medicine.

### Selection of MLS8091-resistant HCMV

MLS8091-resistant HCMV mutants were selected using two different protocols: a multi-step and a single-step approach. The multi-step selection protocol was also used to select for GCV resistance (positive control). In the multi-step selection protocol (33), HFFs were infected with HCMV Towne at a low MOI of 0.01 and treated with half the EC_50_ (½ EC_50_) of MLS8091 or GCV. After complete cytopathic effect (CPE) was observed (2–3 weeks), 5%–10% of the collected supernatant was used to infect fresh cells and to pass the virus at increasing drug concentrations, doubling the drug concentration at each passage. This step was repeated until the virus was passaged in the presence of approximately 10× EC_50_ concentration. Infected (non-drug-treated) cells were maintained in parallel to generate the control WT virus. Drug-resistant viral strains were then plaque-purified three times in the presence of drugs (10× EC_50_), followed by the passage of each viral clone in drug-free conditions for 10 passages. The titer of the resulting drug-resistant viral clones was determined, and stocks were stored at −80°C.

In the single-step selection protocol (34), HFFs were infected with HCMV Towne at an MOI of 0.01 PFU/cell and treated with MLS8091 at a concentration 10× the EC_50_. Drugs were replaced every 5–7 days, and the cells were grown until complete CPE was observed. This step was repeated three times. Infected (non-drug-treated) cells were maintained in parallel as control WT virus. The selected virus was plaque-purified in the presence of drugs (at a 10× EC_50_ concentration), followed by 10 passages in drug-free conditions. The resulting drug-resistant and WT viral clones were titered, and stocks were stored at −80^ᴏ^C.

### DNA sequencing

Next-generation sequencing of HCMV genomes (WT, MLS8091-resistant, and GCV-resistant HCMV Towne) was performed on an Illumina MiSeq (Illumina, San Diego, CA) following 10 passages in drug-free conditions. Starting with ~250 ng of genomic DNA, the samples were sheared by sonication on the Covaris S2 System to a fragment size of approximately 165 bp with the following conditions: duty cycle, 10%; intensity, 5%; cycles/burst, 200; time, 60 s; and number of cycles, 6. Libraries were constructed according to the protocol provided in the Illumina Nano TruSeq Library Prep Kit. Assessment of the yield and size distribution of the amplified library was performed on the Agilent 2100 Bioanalyzer using the High Sensitivity Chip. Libraries were pooled to a final concentration of 2 nM and denatured to a DNA concentration of 20 pM. Denatured DNA was then diluted to a concentration of 10 pM with a 10% PhiX spike to load on an Illumina MiSeq for a 75 × 75 bp paired-end run. Bowtie2 (v2.2.5) was used to align the reads to the HCMV Towne reference. The samtools (v1.5.0) mpileup function was used to compute the coverage of mapped reads on the reference sequence at a single base pair resolution, using default parameters (Table S3). Bcftools (v1.5) view and call functions were used to convert the mpileup file and to call the genomic variants, creating a vcf file. snpEFF and a custom perl script were used to annotate the variant calls. Variant calling was performed using bcftools, a well-validated tool for SNV detection. While alternative variant callers with different sensitivity profiles may identify additional low-frequency variants within viral subpopulations under drug selection, the mutations identified here were subsequently and independently confirmed through marker transfer and functional characterization, establishing their role in MLS8091.

Sanger sequencing was used to confirm specific point mutations generated by *En passant* bacterial artificial chromosome (BAC) mutagenesis protocol. After generating the viral mutants, regions containing point mutations were PCR-amplified, and the resulting PCR products were sequenced to confirm the mutations.

### *En passant* BAC mutagenesis and reconstitution of virus mutants

Markerless introduction of point mutations into HCMV AD169 was achieved using a two-step recombination protocol employing the *en passant* method (35). HCMV AD169-BAC (pAD/Cre) was the backbone for generating point mutations in UL70 and UL102 (36). Briefly, a recombination fragment (PCR product) containing a kanamycin resistance cassette flanked by an I-SceI site (I-SceI-AphAI cassette) from plasmid pEPKan-S was obtained by PCR using primers containing 60 bp homology to the intended integration site in ORF UL70 or UL102 of AD169-BAC and 20 bp specific for pEPKan-S (Table S1); 100 ng of the template DNA (pEPKan-S plasmid) was used. PCR conditions were: 2 min at 95°C, 10× (30 s at 95°C, 30 s at 50°C, and 1 min at 72°C), 25× (30 s at 95°C, 30 s at 60°C, and 1 min at 72°C), 5 min at 72°C, and 10 min at 4°C. All the primers used for *En passant* mutagenesis were PAGE-purified. This fragment was purified using a gel extraction kit (Qiagen, Germantown, MD), and 250 ng of gel-purified DNA was electroporated into *Escherichia coli* GS1783 harboring the AD169-BAC. The first red recombination resulted in a selectable BAC with an ISceI restriction site and a kanamycin resistance cassette flanked by a duplication of the UL70 or UL102 target sequence. After the successful selection of transformants on kanamycin (50 µg/mL) and chloramphenicol (30 µg/mL) plates, all non-HCMV sequences were removed by L-arabinose-induced I-SceI expression and subsequent induction of intrabacterial second red recombination. The second red recombination step resulted in the removal of the kanamycin-resistant cassette and the generation of AD169-BACs with specific mutations in the UL70 or UL102 genes. The point mutations in UL70 and UL102 were confirmed by PCR amplification of the regions flanking these mutations, followed by Sanger sequencing of the PCR products. To reconstitute virus mutants, the recombinant BACs, along with a pp71 expression plasmid, were transfected into HFFs by amaxa Nucleofector kit (program #U021) or electroporation using the Bio-Rad Gene Pulsar Xcell system. Cells were grown until complete CPE was observed, the titer of resulting viral clones was determined, and stocks were stored at −80^ᴏ^C.

### PCR and sequencing of HCMV clinical isolates

Nine wild-type and eight GCV-resistant clinical isolates with known mutations in UL97 were sequenced at the regions of resistant mutations in UL102 and UL70. The primers for UL102 and UL70 are listed in Table S2.

### Statistical analysis

All statistical analyses were performed using the GraphPad Prism software; individual points represent mean ± standard deviation (SD) (*n*  ≥  3). A single-factor analysis of variance (ANOVA) was used to determine the significance of comparing groups, and * represents *P*  ≤  0.05, ** represents *P*  ≤  0.01, *** represents *P*  ≤  0.001, and “ns” represents nonsignificant.

## RESULTS

### HCMV develops resistance to MLS8091

In a recent HTS, we identified five novel structurally distinct inhibitors of HCMV replication. One inhibitor, MLS8091, exhibited an EC_50_ of 0.4 ± 0.01 µM and a full dose-response curve against HCMV Towne (Fig. 1A). Its reported 50% cell viability is >250 µM (29). Although no inhibition of herpes simplex 1 or 2 (HSV-1, 2), or mouse CMV (MCMV), was reported, we found moderate activity of MLS8091 against guinea pig CMV (GPCMV), with EC_50_ values of 3.6 ± 0.4 µM and 3.1 ± 0.1 µM in plaque and PCR assays, respectively (Fig. S1). Similar to GCV, MLS8091 showed reversible inhibition of HCMV, as measured by viral DNA yield on day 6 after compound removal on days 1, 2, or 3 (Fig. 1B).

To investigate the mechanism of action of MLS8091 and determine whether it inhibits HCMV replication by directly targeting a viral protein, we tested whether prolonged exposure to MLS8091 selected for resistant viral progeny. Two independent protocols were employed to generate resistant mutants: a single-step and a multi-step protocol. In the single-step protocol, HFFs were infected with HCMV Towne at an MOI of 0.01 PFU/cell and treated with a 10× EC_50_ concentration of MLS8091 (5 µM). In the multi-step protocol, HFFs were infected with HCMV Towne at an MOI of 0.01 PFU/cell, and the virus was then passaged over time in increasing drug concentrations starting at ½ EC_50_ until 10× EC_50_ concentration was achieved, with a 2-fold increase in drug concentration at each passage (Detailed protocols in Materials and Methods). As expected, wild-type HCMV was completely inhibited by MLS8091 at 3 µM, whereas MLS8091-resistant viral strains were not, indicating that both protocols led to the development of MLS8091 resistance (Fig. 1C and D). The activity of MLS8091 against 8091-resistant HCMV was measured by pp28-luciferase assay. In a parallel experiment, we isolated GCV-resistant HCMV using a multi-step protocol (Fig. 1E). The fold difference in EC_50_ between the MLS8091-resistant and MLS8091-WT Towne viruses was approximately 150 and between the GCV-R and WT viruses was approximately 10 (Table 1 and Fig. 1C, D, and E). Similar to GCV, resistance to MLS8091 developed after 6–7 passages.

**TABLE 1:** Drug selection experiments: design, genotyping, and phenotyping^*a*^

Sample ID	Protocol	Compound	Passages	Seq method	UL70 mutation	UL102 mutation	UL97 mutation	EC_50_ (µM)	Fold-Δ	Fitness vs. WT
SS-8091-R1	SS	MLS8091	3	MiSeq	D487Y	P165S	WT	54.2 ± 11	135.4	No diff.
SS-8091-R2	SS	MLS8091	3	Sanger	D487Y	WT	WT	52.4 ± 14.4	131	No diff.
SS-8091-R3	SS	MLS8091	3	Sanger	D487Y	WT	WT	63.6 ± 14.2	158.9	No diff.
MS-8091-R1	MS	MLS8091	6–7	MiSeq + Sanger	WT	F275L	WT	88.6 ± 2.9	221.6	No diff.
MS-8091-R2	MS	MLS8091	6–7	Sanger	WT	F275L	WT	63.3 ± 6.8	158.2	No diff.
MS-8091-R3	MS	MLS8091	6–7	Sanger	D486H	WT	WT	58.4 ± 10.7	145.9	No diff.
MS-GCV-R1*	MS	GCV	6–7	MiSeq + Sanger	WT	WT	C603W	30 ± 1.1	13.6	N/A
MS-GCV-R2	MS	GCV	6–7	Sanger	WT	WT	C603W	18.4 ± 1.6	8.4	N/A
SS-Control	SS	None	Parallel	Sanger	WT	WT	WT	0.4 ± 0.0	1.0	Ref
MS-Control	MS	None	Parallel	MiSeq + Sanger	WT	WT	WT	0.4 ± 0.01	1.0	Ref

^*a*^ HFFs were infected with HCMV Towne (pp28-luciferase) at an MOI of 0.01 and treated with MLS8091 or GCV. As described in the Materials and Methods, SS and MS resistance selection protocols were used. EC_50_ values: mean ± SD (≥ 3 independent experiments). *GCV WT EC_50_: 2.2 ± 0.23 µM. N/A, not assessed.

### Identification of mutations in MLS8091-resistant HCMV

To identify the viral targets responsible for MLS8091 resistance, next-generation sequencing (NGS) of WT, MLS8091-resistant, and GCV-resistant viral strains was performed using the Illumina MiSeq platform (Table S3). Point mutations in the HCMV primase (UL70) and primase-associated factor (UL102) were identified in MLS8091-resistant but not in WT or GCV-resistant HCMV (Table 1). MLS8091-resistant strains isolated using the SS resistance selection protocol harbored a single point mutation in HCMV UL70 (UL70 1459G > T, D487Y), as shown in Fig. S2. One strain harbored the same UL70 point mutation, along with a single point mutation in the UL102 gene (UL70 1459G > T, D487Y + UL102 493C>T, P165S), as shown in Fig. S2. Two viral strains, selected using the MS protocol, harbored a single point mutation in UL102 (UL102 823T > C, F275L), while one clone was found to have a single point mutation in UL70 (UL70 1456 G > C, D486H) (Table 1 and Fig. S2). GCV-resistant HCMV strains harbored a previously reported UL97 point mutation (1809 C > G, C603W) (15, 37, 38). No mutations were identified in the CMV helicase, UL105.

NGS and Sanger sequencing identified the following mutations: UL70 D486H, D487Y, and UL102 P165S, F275L. Mutations in UL102 were identified in resistant strains isolated by SS (P165S) and the MS-resistant selection protocols (F275L), but not in the WT HCMV (passaged in drug-free conditions) or GCV-resistant HCMV strain. Viral strains selected using the SS protocol also harbored a point mutation in UL70 D487Y (Table 1 and Fig. S2). The HCMV UL70 and UL102 point mutations were confirmed in additional MLS8091-resistant strains using Sanger sequencing of the UL70 and UL102 regions. A mutation in UL97 was also confirmed and was found in another GCV-resistant HCMV clone (Table 1 and Fig. S2D).

### HCMV Towne strain that harbors point mutations in UL70 (D487Y and D486H) is resistant to inhibition by MLS8091

**Fig 2:**
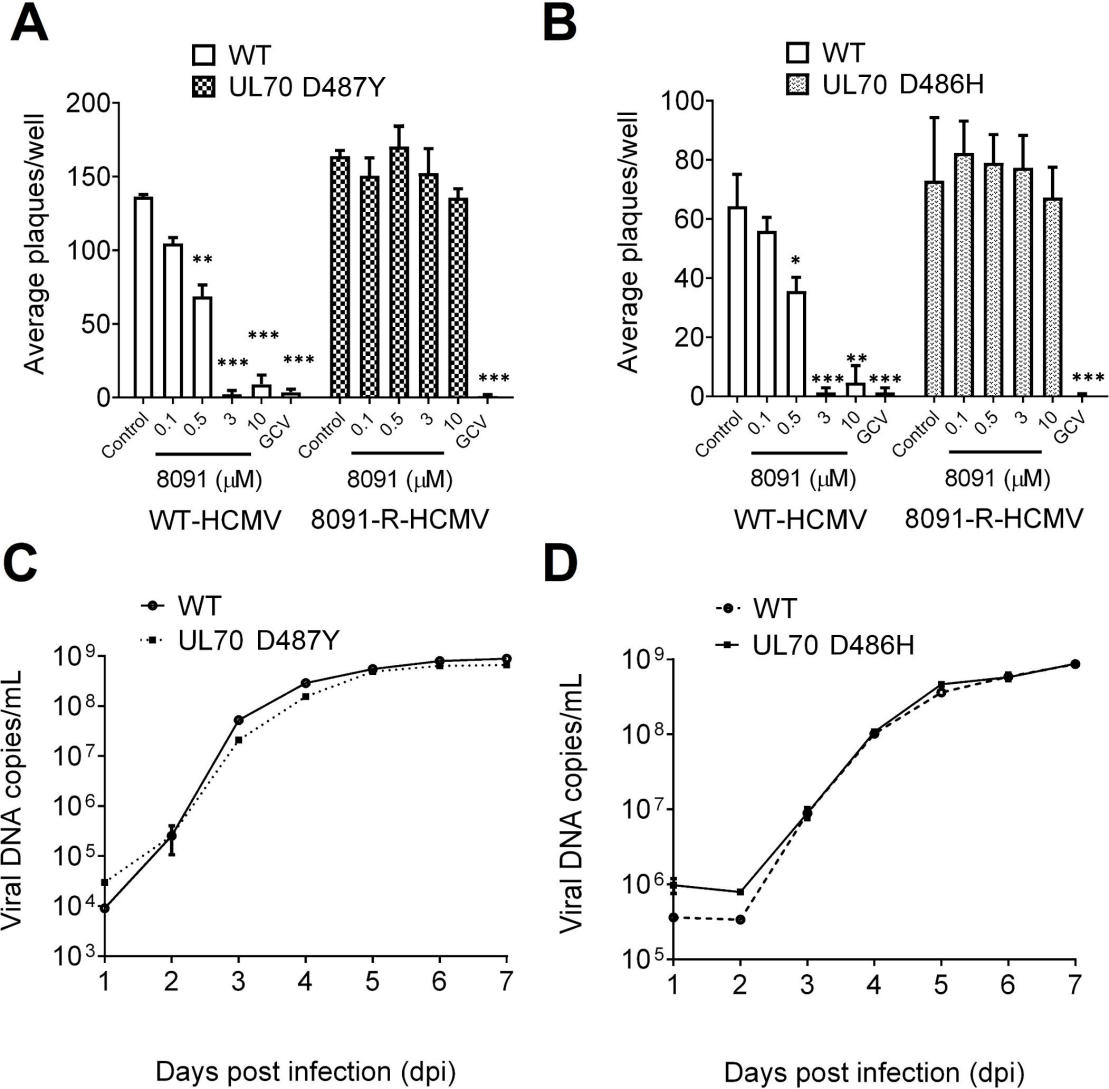
HCMV Towne, which harbors mutations in UL70, is not inhibited by MLS-8091**.** (**A**) HFFs were infected with 150 PFUs of WT or MLS8091-resistant HCMV Towne SS-8091-R3 (UL70 D487Y) and treated with the indicated concentrations of MLS8091. Plaques were stained and counted after 10 days. Statistical significance is assessed against the infection-only (control) condition. (**B**) HFFs were infected with 100 PFUs of WT or MLS8091-resistant HCMV Towne, SS-8091-R3 (UL70 D486H), and treated with the indicated concentrations of MLS8091. Plaques were stained after 10 days. (**C**) HFFs were infected with WT or MLS8091-resistant HCMV Towne (UL70 D487Y) at an MOI of 0.1, and supernatants were harvested 1–7 days post-infection. Viral DNA load was quantified using real-time PCR for US17. (**D**) HFFs were infected with WT or MLS8091-resistant HCMV Towne (UL70 D486H) at an MOI of 0.1, and supernatants were harvested 1–7 days post-infection. Viral DNA load was determined by real-time PCR for US17.

We first tested the activity of MLS8091 against HCMV strains that harbored mutations in the HCMV primase UL70 (D487Y) or (D486H). HFFs were infected with the WT-Towne strain or UL70 D487Y-HCMV Towne (Fig. 2A), and the activity of MLS8091 was measured by plaque assay. A dose-dependent decrease in plaque numbers was observed for WT HCMV, while the HCMV Towne UL70 D487Y was not inhibited by MLS8091 even at 10 µM (Fig. 2A), suggesting that this point mutation was sufficient to confer resistance to MLS8091. Similarly, the effect of the UL70 D486H mutation on MLS8091 activity was tested. HFFs were infected with WT-HCMV Towne or HCMV Towne containing the UL70 D486H mutation, and the activity of MLS8091 was tested by a plaque reduction assay. As observed for the UL70 1459 G > T point mutation, HCMV Towne with UL70 D486H mutation was not inhibited by MLS8091 (Fig. 2B), while the WT-HCMV Towne was sensitive to MLS8091 treatment, suggesting that UL70 D486H mutation was also sufficient to confer resistance to MLS8091. Growth curve analysis was performed to compare the growth kinetics of WT and mutant Towne viruses; no significant difference was observed, indicating that neither of the HCMV UL70 point mutations affected the fitness of the mutant HCMVs (Fig. 2C and D).

### A point mutation in AD169 UL70 (D487Y) confers resistance to MLS8091

**Fig 3:**
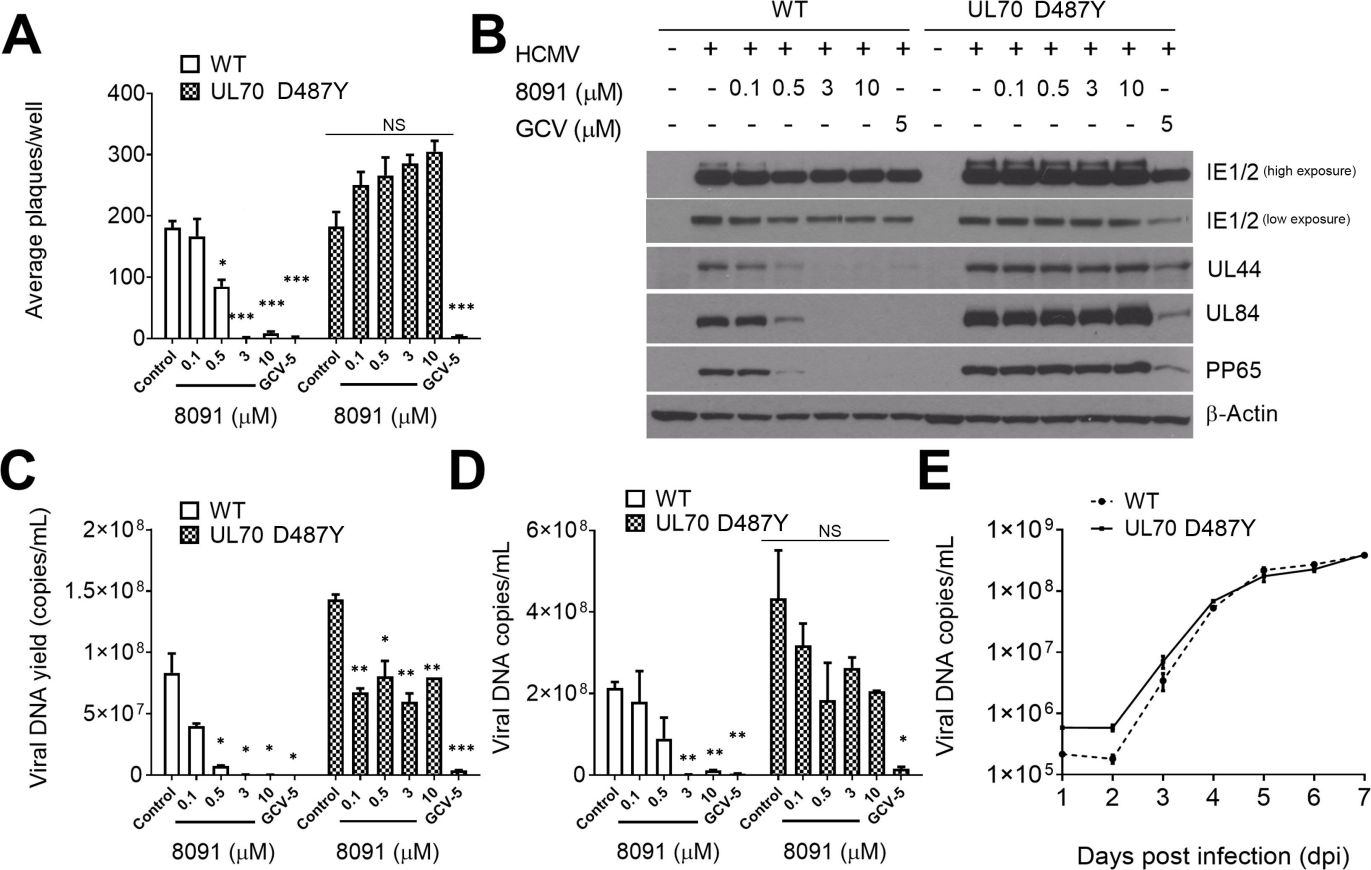
The point mutation in AD169 UL70 D487Y confers resistance to MLS8091**.** (**A**) HFFs were infected with 200 PFUs of WT- or UL70 D487Y, and the activity of MLS8091 was measured by plaque reduction assay. (**B**) HFFs were infected with WT- or UL70-D487Y AD169 (MOI 0.1), and the expression of viral proteins IE1/2, UL44, UL84, and pp65 was measured 4 days post-infection. (**C**) HFFs were infected with WT- or UL70-D487Y AD169 (MOI 0.1), and viral load in the supernatants was measured by US17 real-time PCR at 4 days post-infection. (**D**) HFFs were infected with WT- or UL70-D487Y AD169 (MOI 0.1), and viral DNA was quantified in infected cell extracts at 48 hpi by US17 real-time PCR. NS- non-significant. (**E**) Growth Curve assay: HFFs were infected with WT AD169 or UL70 D487Y AD169 (MOI 0.1). Supernatants were collected post-infection from days 1 to 7, and viral load was quantified using US17 real-time PCR. Statistical significance is compared with the infected-only (control) group.

To confirm that the point mutations in the HCMV primase UL70 identified by NGS were *specifically and directly* responsible for MLS8091 resistance, we introduced these mutations into the HCMV AD169 strain by *En passant* mutagenesis using the AD169 BAC. Following mutagenesis, AD169 virus particles were reconstituted by electroporation of AD169 BACs into HFFs. We measured the activity of MLS8091 against WT, UL70 D487Y, and AD169 using a plaque reduction assay. While WT AD169 showed a clear, dose-dependent reduction in plaque numbers, with 20-fold to 100-fold inhibition at 3 and 10 µM MLS8091, the UL70 D487Y mutant exhibited no inhibition (Fig. 3A). Although the mutant showed 1.5-fold to 1.7-fold increase in plaque numbers at the higher drug concentrations, there was no statistically significant difference between any MLS8091-treated group and the untreated control for UL70 D487Y (*P* > 0.05 for all comparisons). This minor fluctuation is biologically insignificant when compared to the >20-fold inhibition observed in the WT and is consistent with normal inter-well variability in plaque assays. Additionally, GCV retained full activity against UL70 D487Y, confirming that the resistance phenotype is specific to MLS8091. Additional plaque assays using MLS8091 at higher concentrations (up to 100 mM) resulted in an EC_50_ of AD169 UL70 to MLS8091 of 40.84 ± 1.71 EC_50_. The fold difference in EC_50_ between the MLS8091-resistant and MLS8091-WT AD169 was 97.3 (Table 2).

**TABLE 2:** Marker transfer AD169-BAC: Genotype and Phenotype

Recombinant (AD169-BAC)	UL70 genotype	UL102 genotype	MLS8091 EC_50_ (µM)	Fold-Δ vs. WT	GCV susceptible	
WT-AD169	WT	WT	0.42 ± 0.10	1.0× (reference)		Yes
UL70 D487Y	D487Y	WT	40.84 ± 1.71	97.3		Yes (Fig. 3B)
UL70 D486H	D486H	WT	41.05 ± 1.36	97.8		Yes (Fig. 4B)
UL102 F275L	WT	F275L	53.57 ± 14.99	127.6		Yes (Fig. 6B)
UL70 D487Y + UL102 P165S	D487Y	P165S	40.28 ± 1.42	95.9		Yes (Fig. 7B)
UL102 A294G	WT	A294G	0.72 ± 0.03	1.7		Yes

**Fig 4:**
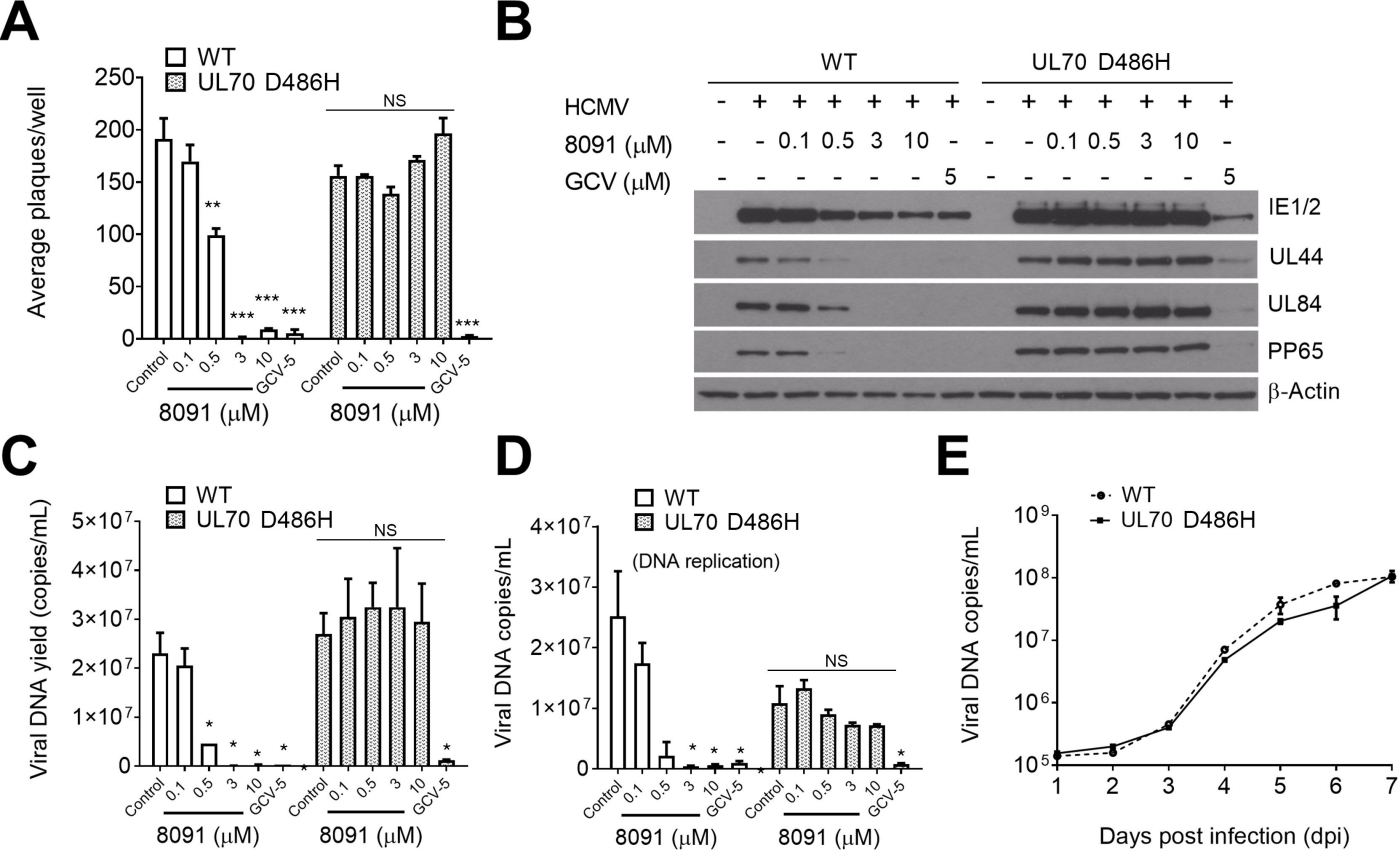
Point mutation in HCMV UL70 D486H confers resistance to MLS8091. (**A**) HFFs were infected with 200 PFUs of WT- or UL70 D486H AD169, and the activity of MLS8091 was measured by plaque reduction assay. (**B**) HFFs were infected with WT- or UL70 D486H AD169 (MOI 0.1), and the expression of viral proteins was measured 4 days post-infection. (**C**) HFFs were infected with WT- or UL70-D486H AD169 (MOI 0.1), and viral load in the supernatants was determined 4 days post-infection by US17 real-time PCR. (**D**) HFFs were infected with WT- or UL70-D486H AD169 (MOI 0.1), and viral DNA replication was quantified at 48 hpi by US17 real-time PCR. (**E**) HFFs were infected with WT AD169 or AD169 with a single point mutation in UL70 (D486H). Supernatants were collected post-infection from days 1 to 7, and viral load was quantified using US17 real-time PCR. Statistical significance is assessed against the infection-only (control) condition. NS- non-significant.

Next, we determined the effect of MLS8091 on HCMV AD169 viral protein expression. HFFs were infected with the WT- and UL70 D487Y mutant AD169 (MOI 0.1) and treated with various concentrations of MLS8091. Viral protein expression was determined by Western blots at 96 hpi (Fig. 3B). While a dose-dependent inhibition of CMV proteins IE1/2, UL44, UL84, and pp65 was observed in WT AD169 treated with MLS8091, there was no decrease in these viral proteins with the UL70 D487Y strain treated with MLS8091, indicating this mutation was sufficient to confer resistance to MLS8091. MLS8091 exhibited reduced antiviral activity against AD169 UL70 D487Y compared to WT AD169. While MLS8091 significantly inhibited viral load in supernatants from WT AD169 in a dose-dependent manner (Fig. 3C, left panel), a statistically significant but substantially attenuated inhibitory effect was observed against UL70 D487Y (Fig. 3C, right panel). Similarly, MLS8091 significantly inhibited viral DNA quantification in infected cell extracts from WT AD169 at 48 hpi (Fig. 3D, left panel), while the inhibitory effect on UL70 D487Y was not statistically significant (Fig. 3D, right panel).

The growth kinetics of WT-AD169 and UL70 D487Y AD169 were measured by US17 real-time PCR from days 1 to 7 post-infection. There was no difference in viral growth, suggesting that the D487Y mutation did not alter the viral fitness (Fig. 3E).

### A point mutation in UL70 D486H AD169 confers resistance to MLS8091

The same procedure was performed to test whether the D486H mutation in HCMV UL70 confers resistance to MLS8091. The activity of MLS8091 against WT- and UL70 D486H AD169 was measured by plaque reduction assay (Fig. 4A and Table 2), viral protein expression (Fig. 4B), viral load in supernatants (Fig. 4C), and viral DNA replication (Fig. 4D). In all the assays, UL70 D486H AD169 conferred resistance to MLS8091. Similar to the D487Y mutant strain (Fig. 3 and Table 2), the D486H strain was 97.8-fold resistant to MLS8091 compared to AD169 WT (Table 2). UL70 D486 had no effect on viral growth, suggesting that it did not alter viral fitness (Fig. 4E).

### HCMV Towne, which harbors a UL102 mutation (F275L), is not inhibited by MLS8091

**Fig 5:**
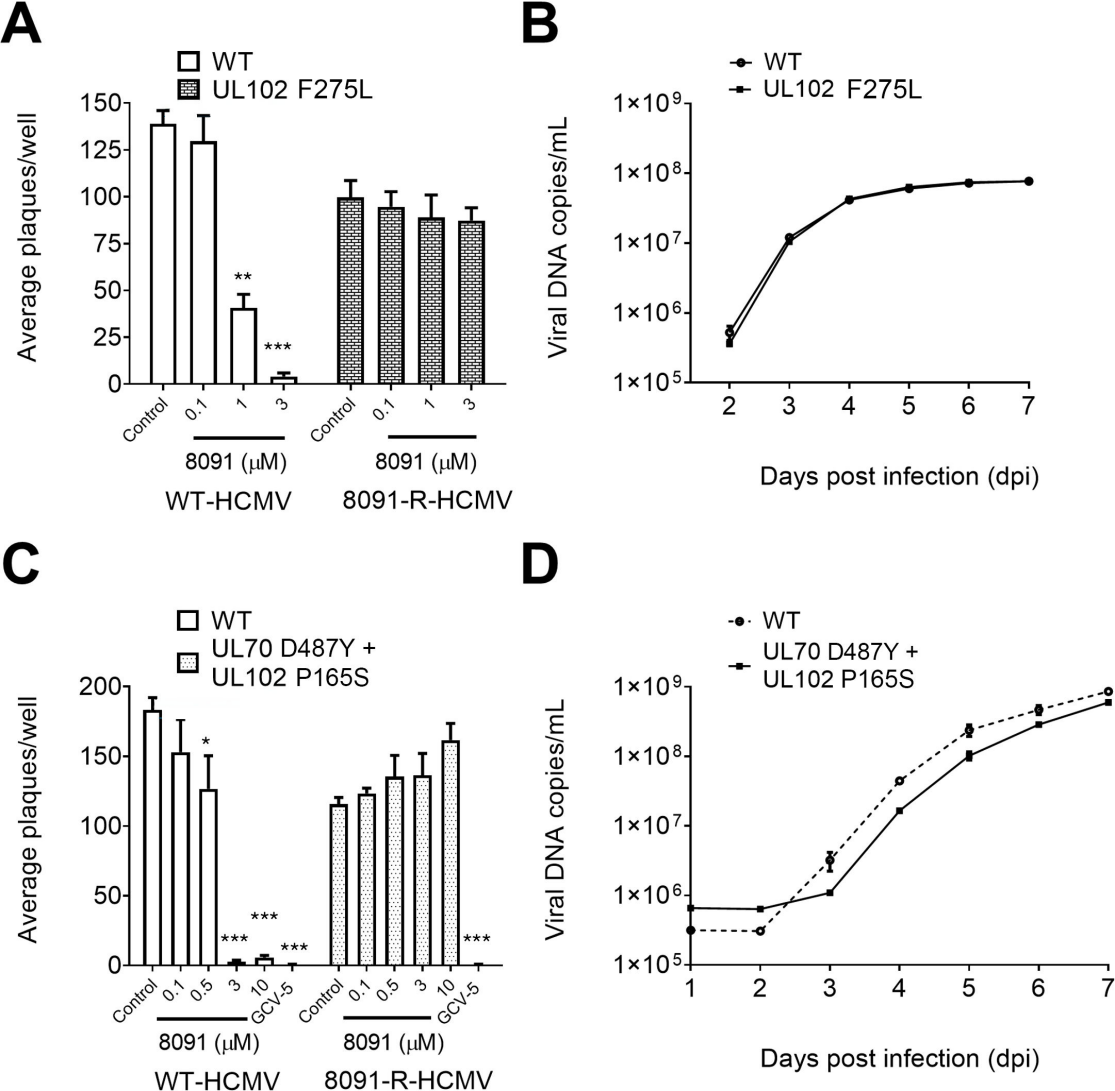
HCMV Towne carrying UL102 F275L or UL70 D487Y, together with UL102 P165S, is resistant to MLS8091. (**A**) HFFs were infected with 200 PFUs/well of WT or UL102 F275L (MS-8091-R1) HCMV Towne and treated with the indicated concentrations of MLS8091. Plaques were stained and counted at day 10 post-infection. (**B**) HFFs were infected with WT or UL102 (F275L, MS-8091-R1) HCMV Towne (MOI 0.1), supernatants were collected from day 1 to day 7 post-infection, and viral DNA load was quantified by US17 real-time PCR. (**C**) HFFs were infected with 200 PFUs/well of WT or UL102 P165S + UL70 D487Y HCMV Towne (SS-8091-R-1) and treated with the indicated concentrations of MLS8091. Plaques were stained and counted at day 10 post-infection. (**D**) HFFs were infected with WT or UL102 P165S + UL70 D487Y HCMV Towne SS-8091-R-1 (MOI 0.1), supernatants were collected at days 1–7 post-infection, and viral DNA load was quantified by US17 real-time PCR. Statistical significance is compared to infection only (control).

NGS of two MLS8091-resistant viral mutants obtained using the multi-step (MS) resistance protocol identified the point mutation F275L in the HCMV primase-associated factor UL102 (Table 1). One of the MLS8091-resistant viral mutants obtained using the SS resistance protocol exhibited point mutations in the HCMV primase gene, UL70 (D487Y), and in the HCMV primase-associated factor gene, UL102 (P165S) (Table 1). We determined the effects of these mutations on MLS8091 activity and on HCMV growth. The UL102 F275L single mutant and the UL70 D487Y plus UL102 P165S double mutant in HCMV Towne were both resistant to MLS8091 (Fig. 5A and C). There was no difference in HCMV growth with these mutations compared to the WT virus (Fig. 5B and D).

### A point mutation in AD169 UL102 (F275L) confers resistance to MLS8091

**Fig 6:**
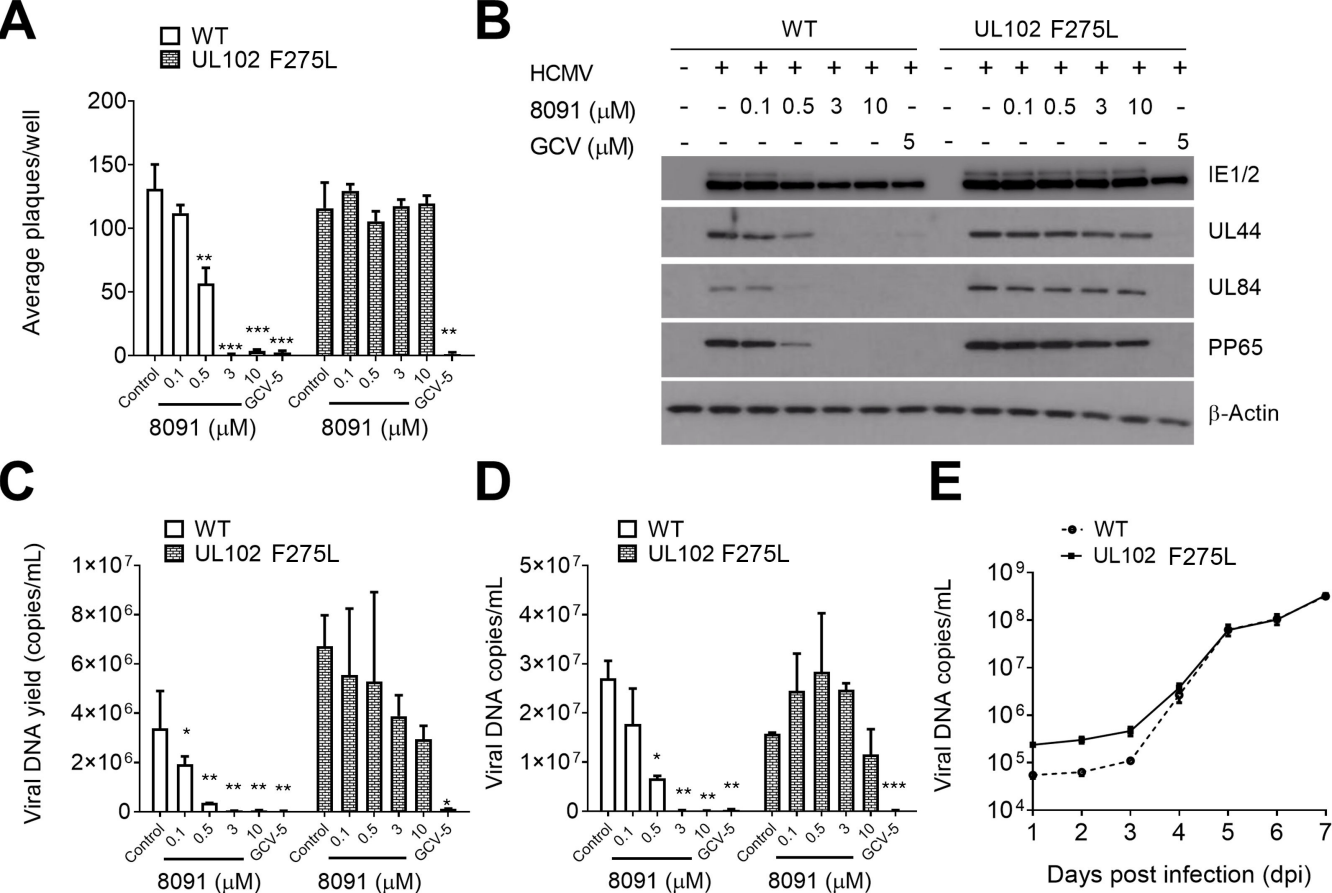
Point mutation in UL102 (F275L) confers resistance of AD169 to MLS8091. (**A**) HFFs were infected with 150 PFUs of WT- or UL102 F275L AD169, and the activity of MLS8091 was measured by plaque assay at 10 days post-infection. (**B**) HFFs were infected with WT- or UL102 F275L AD169 (MOI 0.1), and the expression of viral proteins was measured 4 days post-infection. (**C**) HFFs were infected with WT- or UL102 F275L AD169 (MOI 0.1), and viral DNA load in the supernatants was measured by US17 real-time PCR at 4 days post-infection. (**D**) HFFs were infected with WT- or UL102 F275L AD169 (MOI 0.1), and viral DNA replication was quantified by US17 real-time PCR. (**E**) HFFs were infected with WT AD169 or UL102 F275L AD169 (MOI 0.1), supernatants were collected from day 1 to day 7 post-infection, and viral load was quantified by US17 real-time PCR.

We next generated AD169 with the specific UL102 mutation F275L and tested MLS8091 activity. UL102 F275L conferred resistance to MLS8091, shown by plaque reduction assay (Fig. 6A), viral protein expression at 4 days post-infection (Fig. 6B), viral load in supernatants (Fig. 6C), and viral DNA replication at 48 hpi (Fig. 6D), confirming that this single point mutation in the HCMV primase-associated factor was sufficient for resistance generation. Compared to WT AD169, there was no alteration in the growth kinetics of the mutant HCMV AD169 (Fig. 6E) .

**Fig 7:**
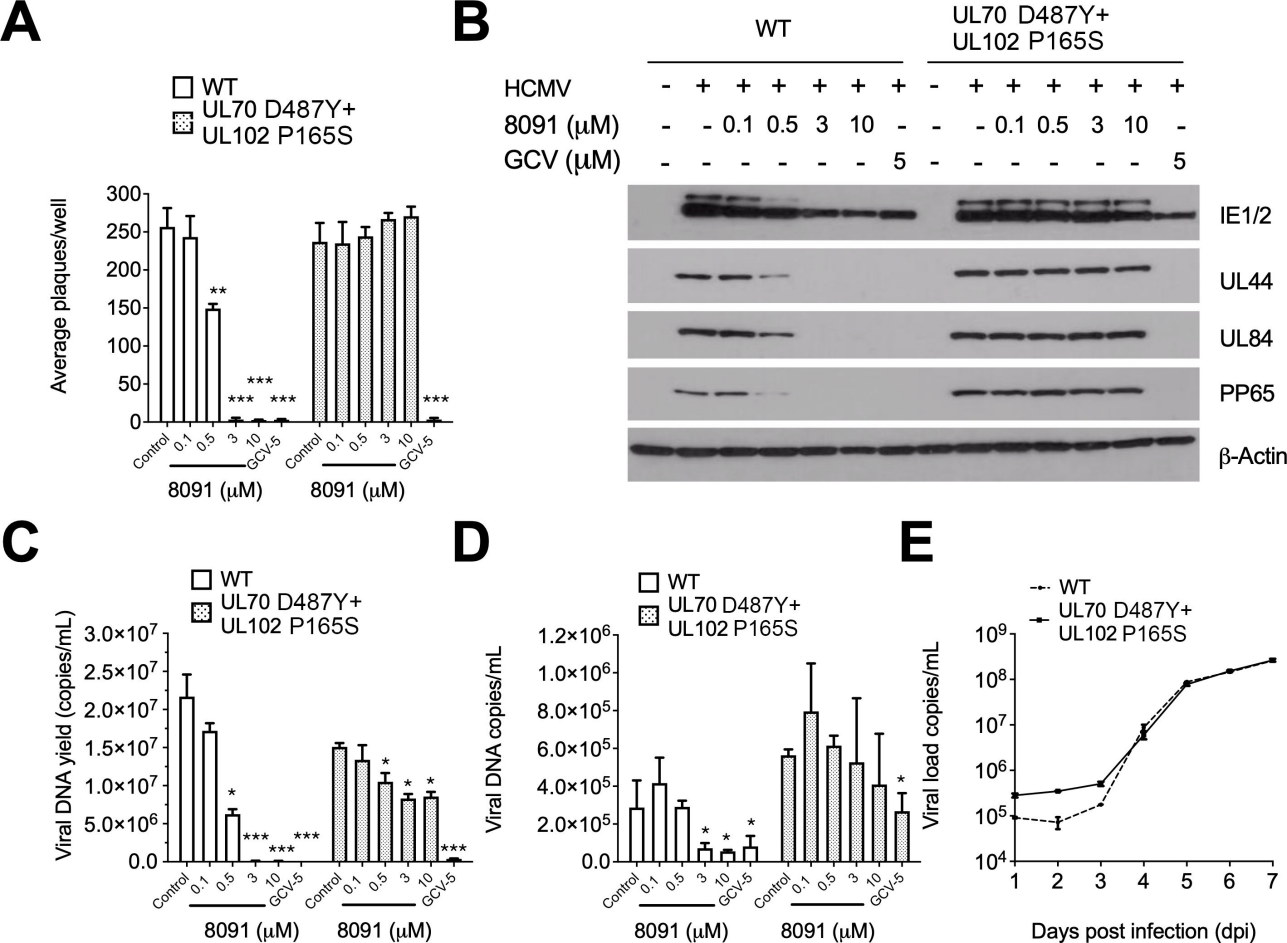
AD169 carrying UL70 D487Y together with UL102 P165S is resistant to MLS8091. (**A**) HFFs were infected with 200 PFUs of WT- or UL102 P165S + UL70 D487Y AD169, and the activity of MLS8091 was measured at day 10 post-infection by plaque reduction assay. (**B**) HFFs were infected with WT- or UL102 P165S + UL70 D487Y AD169 (MOI = 0.1), and the expression of viral proteins was measured at 4 days post-infection. (**C**) HFFs were infected with WT- or UL102 P165S + UL70 D487Y AD169 (MOI 0.1), and viral DNA load in supernatants was measured by US17 real-time PCR at day 4 post-infection. (**D**) HFFs were infected with WT- or UL102 P165S + UL70 D487Y AD169 (MOI 0.1), and viral DNA replication was quantified by US17 real-time PCR at 48 hpi. (**E**) HFFs were infected with WT- or UL102 P165S + UL70 D487Y AD169 (MOI 0.1), supernatants were collected at days 1–7 post-infection, and viral load was quantified by US17 real-time PCR. Statistical significance compared to infection only (control).

We next generated AD169 with mutations in UL70 (D487Y) and UL102 (P165S). AD169 UL70 D487Y was used as the backbone to create the UL102 P165S mutation. The double mutant AD169 was not inhibited by MLS8091, as determined by plaque assay (Fig. 7A), protein expression (Fig. 7B), viral load in supernatants (Fig. 7C), and viral DNA replication (Fig. 7D). Because the P165S substitution was generated in a UL70 D487Y background, and the double mutant displayed an EC_50_ and fold-resistance were similar to UL70 D487Y alone (Table 2), the contribution of P165S to MLS8091 resistance could not be determined independently. Statistical significance was assessed relative to the infection-only (control) group.

### Polymorphisms in UL70 and UL102 in clinical isolates

**Fig 8:**
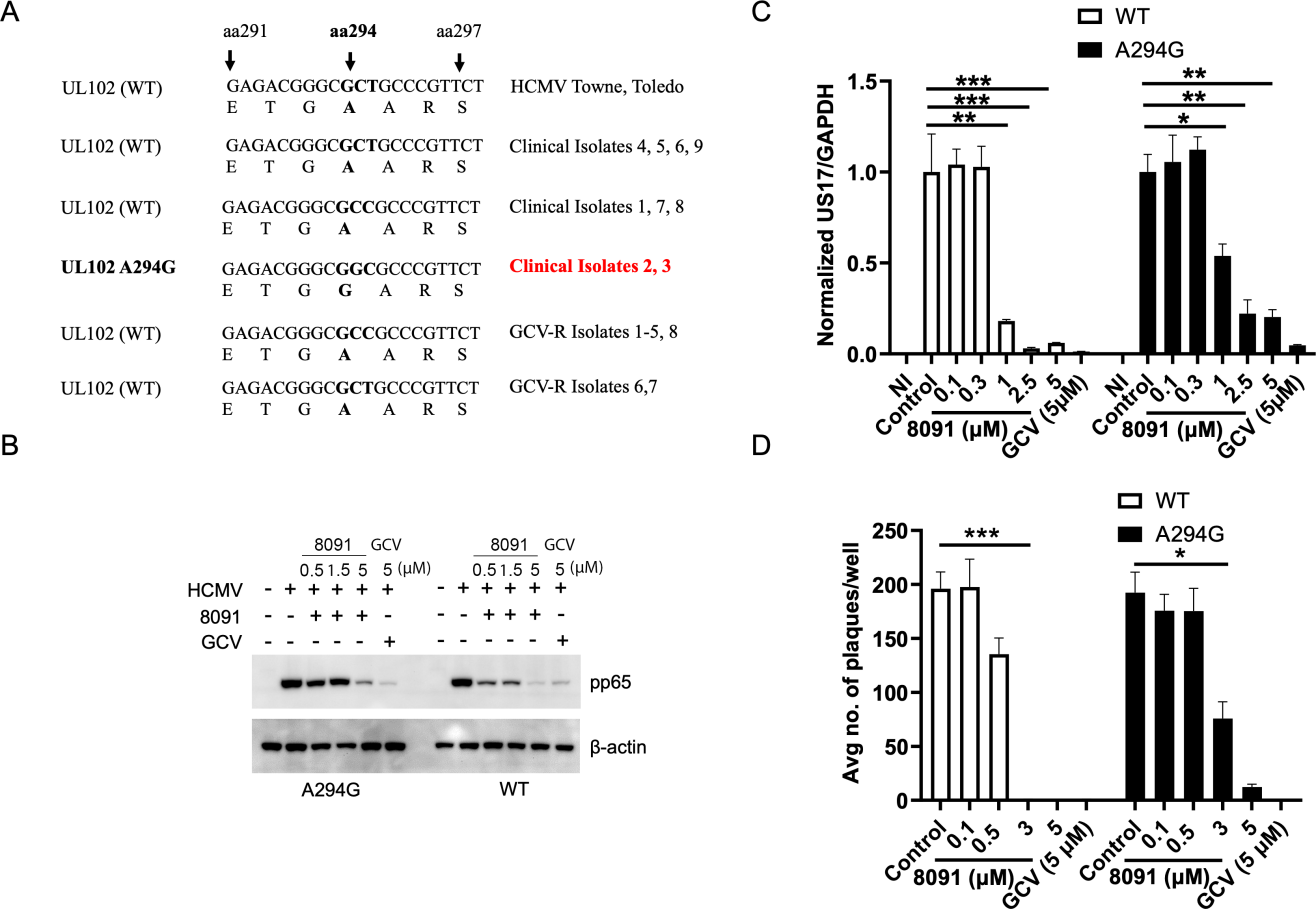
A UL102 A294G polymorphism is present in clinical isolates that show reduced susceptibility to MLS8091 but does not confer MLS8091 resistance upon marker transfer. (**A**) The specific mutations in UL102 were sequenced from nine WT and eight GCV-R clinical isolates. Two of eight clinical isolates contained a new polymorphism (A294G). (**B**) HFFs were infected with clinical isolates (UL102 WT and mutant) at an MOI of 0.5 and treated with different concentrations of MLS8091 for 80 h. The cells were lysed, and an immunoblot was performed for pp65 and β-actin. GCV treatment was used as a positive control. (**C**) A similar experiment was performed as in A, except the cells were harvested at 60 h post-infection; DNA was isolated, and real-time PCR was performed for HCMV-US17 and GAPDH. The graph shows normalized US17/GAPDH ratios from three independent experiments. * represents *P* ≤ 0.05, ** *P* ≤ 0.01, and *** *P* ≤ 0.001; 1, 2.5, and 5 μM of MLS8091 treatment conditions were compared to infected control for both WT and Ala294Gly. (**D**) HFFs were infected with clinical isolates with or without UL102 mutation (200 PFU/well) and treated with various concentrations of MLS8091 diluted in DMEM containing methylcellulose. The plates were stained with crystal violet after 9 days, and plaques were counted. The average plaque numbers/well are representative of a single experiment among three independent experiments. * represents *P* ≤ 0.05, *** *P* ≤ 0.001; 3 μM of MLS8091 treatment condition was compared to the infected control for both WT and Ala294Gly.

We evaluated whether HCMV clinical isolates harbored preexisting mutations in the primase or primase-associated factor. Nine WT clinical isolates and eight GCV-resistant isolates were sequenced using primers that covered the regions of identified mutations in UL102 and UL70 (Table S2). The MLS8091-selected mutations were not found in clinical isolates (Figs. S3 and 4). A new polymorphism in UL102, Ala294Gly, was identified in two of nine WT clinical isolates, suggesting that it may also be involved in the mechanism of MLS8091 (Fig. 8A and chromatograms in Figs. S3 and 4). We tested the activity of MLS8091 against a WT clinical isolate without any identified mutations in the H/P, and against a clinical isolate harboring the Ala294Gly polymorphism. While the WT clinical isolate was inhibited by MLS8091, the mutant clinical isolate showed reduced susceptibility to MLS8091, as indicated by western blot (Fig. 8B), US17 real-time PCR (Fig. 8C), and plaque assay (Fig. 8D). However, marker transfer of A294G into AD169-BAC resulted in an EC_50_ of 0.72 μM compared to 0.42 μM for WT (1.7-fold), indicating the lack of clinically meaningful antiviral resistance, and suggesting other viral determinants account for the phenotype observed in A294G-harboring isolates (Table 2).

### Comparison of amino acid sequences in HCMV, HSV-1, MCMV, and GPCMV primase and primase-associated factor

Although low sequence similarity was observed in the region of MLS8091-selected resistance among the virus strains, GPCMV showed sequence similarity at UL70 amino acids 486/487 and UL102 amino acid 166 (Fig. S5), which may explain its moderate inhibition by MLS8091. In addition, several residues flanking amino acid 486 (1–3) are shared between HCMV and GPCMV but diverge in MCMV. These differences in the residues surrounding conserved residue 486 may alter the folding of the binding pocket, preventing MLS8091 binding to MCMV despite conservation of the key aspartate. The modest activity of MLS8091 against GPCMV, which shares more of the local context with HCMV than with MCMV, is consistent with this interpretation.

## DISCUSSION

We report a comprehensive investigation of a novel HCMV target using the small molecule MLS8091. Approximately two decades ago, an HCMV inhibitor, T-0902611, was reported to covalently modify the HCMV primase (39). A subsequent study of the imidazolyl-pyrimidine series reported that the compounds were irreversible inhibitors of UL70, based on radiolabeling and SAR, but no resistance studies were performed (40). We report here on a small molecule that specifically and directly inhibits the HCMV H/P complex, representing a mechanistically distinct class of anti-HCMV agents. Through resistance selection and marker transfer experiments, we demonstrate that point mutations in either the viral primase (UL70, amino acids 486–487) or the primase-associated factor (UL102, amino acid 275) confer resistance to MLS8091, thereby establishing the H/P complex as the molecular target responsible for antiviral activity. Since the HCMV H/P complex is crucial for viral DNA replication, it is a highly attractive target for antiviral therapy.

The identification of resistance mutations in two separate components of the H/P complex provides insight into the potential mechanism of action. The observation that mutations in either UL70 or UL102 independently confer resistance suggests that MLS8091 may act at the interface between these two proteins or disrupt a functional interaction that requires both components. We propose that MLS8091 may require this interface for its inhibitory activity and that resistance mutations restore viral replication by reducing the compound’s ability to bind its target. The mutations in UL102 are not close to each other. Despite their adjacent positions in UL70, D486H and D487Y may project into different local microenvironments due to alternating side-chain orientations, with D486H making more direct contact with the MLS8091 binding site. Experimental structural characterization of the UL70-UL102 complex, ideally in the presence of MLS8091, will be an important future direction for understanding the drug-binding mode and informing the development of next-generation helicase-primase inhibitors. Notably, no resistance mutations were identified in UL105, the helicase component of the complex, suggesting that MLS8091 preferentially targets the primase-associated functions rather than helicase activity *per se*. It is possible that the primase and primase-associated factor accommodate substitutions at the drug-contact residues because the primase catalytic mechanism tolerates peripheral surface changes better than the helicase’s tightly coupled ATPase-unwinding mechanism. The HSV helicase-primase inhibitor pritelivir binds at the HSV helicase-primase interface, predominantly on the helicase side (41). It appears that MLS8091 places its critical contacts on the primase-primase-associated factor side.

The HCMV H/P complex plays an essential role in viral DNA replication. The helicase unwinds double-stranded DNA at the replication fork, while the primase synthesizes RNA primers for lagging-strand synthesis. The three components of the complex, UL105 (helicase), UL70 (primase), and UL102 (primase-associated factor), form a functional unit in which each protein contacts the others, and the activities of the helicase and primase are mutually stimulatory (42).

Several classes of HSV H/P inhibitors have been developed, including the 2-aminothiazoles, thiazolylphenyl compounds (BILS 179 BS), thiazoleamides (BAY 57-1293/pritelivir), and oxadiazolephenyl derivatives (ASP2151/amenamevir) (43–47). Amenamevir inhibits HSV-1 and 2 and varicella (VZV) (48, 49). Its oral formulation was more effective in mice than valacyclovir in reducing intradermal HSV-1 titers and treating HSV keratitis (50). In a Phase II study, amenamevir was found to be safe and effective for genital HSV-2 (46), and it is currently in Phase III clinical trials (NCT01959295). Pritelivir has received FDA fast-track designation. A recent study of H/P inhibitor IM-250 in an intravaginal GP model revealed that latency can also be treated, resulting in a significant reduction in reactivations (51). However, none of these compounds demonstrates activity against HCMV, despite presumed functional conservation of the H/P complex across herpesviruses. The lack of cross-activity may reflect sequence divergence between the HSV and HCMV H/*P* proteins; UL70 shares only 28.7% amino acid identity with its HSV-1 homolog UL52, and UL102 shares 28.6% identity with HSV-1 UL8. The discovery of MLS8091, which inhibits HCMV but not HSV, provides a complementary tool for dissecting the unique biology of the HCMV H/P complex.

The HCMV H/P proteins were thought to have sequence homology and positional similarities to the HSV-1 H/P proteins UL5, UL52, and UL8, respectively (52). They are of great interest as potential new drug targets because of their pivotal role in DNA synthesis. UL70 encodes a 947 amino acid (aa) protein, 107 kDa, that, like the HSV-1 primase, contains a putative DXD motif common to the metal-binding site in prokaryotic primases (52). HCMV UL105 encodes a 982 aa, 109 kDa protein that, like HSV-1 UL5, contains six conserved helicase motifs common to the superfamily-1 DNA helicases (53). HCMV UL102 was initially predicted to encode a 798 aa, 85 kDa protein with limited sequence similarity to HSV-1 UL8 (52). Later, an unspliced 2.7 kb transcript from infected cells was found to encode a 100 kDa protein; UL102 was resequenced, confirming a single open reading frame (54). The HCMV H/P complex was only studied in transfected cells using recombinant expression systems. All three proteins formed a complex, and each member was in contact with every other protein (55). An interaction between UL70-UL105 and UL70-UL102 was demonstrated (55).

Using sequence alignment of herpesvirus homologs, conserved amino acids were identified in the UL105 ATP binding site and the pUL70 zinc finger domain. Mutational analysis of several of these amino acids in UL105 and UL70 showed that they are crucial for HCMV replication (56). We note that MLS8091 did not select for resistance in UL105 during the resistance selection experiments; however, its exact binding site is yet to be determined.

Among the herpesviruses tested, only GPCMV showed moderate susceptibility (EC_50_ approximately 3.5 µM compared to 0.4 µM for HCMV), while HSV-1, HSV-2, and MCMV were not inhibited. Sequence alignment revealed that GPCMV shares sequence identity with HCMV at UL70 amino acids 486–487 and UL102 amino acid 165, positions implicated in MLS8091 resistance. This partial conservation may explain the intermediate susceptibility of GPCMV. The moderate activity against GPCMV raises the possibility that this animal model, which has proven valuable for studying congenital CMV infection and vaccine development, could potentially be adapted for *in vivo* evaluation of MLS8091 analogs (57). However, efficacy would need to be demonstrated, given the reduced potency.

Our study reveals that MLS8091 retains activity against GCV-resistant HCMV strains. Drug-resistant HCMV represents a growing clinical challenge, particularly in transplant recipients who require prolonged antiviral therapy. Current treatment options for resistant HCMV are limited; foscarnet and cidofovir are associated with significant nephrotoxicity, and cross-resistance between these DNA polymerase inhibitors can occur (12, 13, 15, 37). While letermovir and maribavir have expanded the therapeutic armamentarium, resistance to these agents has already been reported (19, 24). An inhibitor targeting the H/P complex would provide a mechanistically orthogonal option for patients with resistant HCMV. The fact that MLS8091-resistant mutants remained susceptible to GCV suggests that combination or sequential therapy could be developed to suppress the emergence of resistance.

Finally, beyond acquired resistance, we also evaluated whether preexisting mutations might affect susceptibility to MLS8091. Although we identified a UL102 polymorphism in 2/17 clinical isolates, it showed similar susceptibility to MLS8091 in marker transfer experiments, suggesting that, in this collection of clinical isolates, there were no pre-existing H/P resistance mutations. Among 30 clinical isolates of HSV-1, all susceptible to the H/P inhibitor BAY 57-1293, five had preexisting mutations in the HSV-1 helicase (58). The clinical significance of polymorphisms in the HCMV H/P will depend on their prevalence in a larger number of HCMV strains and the degree to which they compromise antiviral efficacy.

In summary, we have identified MLS8091 as a novel inhibitor of HCMV replication that acts through the helicase-primase complex, a target not exploited by any currently approved anti-HCMV therapy. The compound is active against GCV-resistant strains and exhibits high selectivity for HCMV over other herpesviruses. These findings open new avenues for the development of HCMV-specific antiviral agents that could address the unmet clinical need for safe and effective therapies, particularly for drug-resistant infections and congenital CMV disease. Future studies should focus on determining the binding site and mechanism of action of MLS8091. Evaluation in animal models, potentially including the GPCMV model, will be important for assessing *in vivo* efficacy and safety.

## SUPPLEMENTAL MATERIAL

Supplemental figures aac.01977-25-s0001.pdf Fig. S1 to S5.

Supplemental material aac.01977-25-s0002.docx Supplemental figure legends.

Supplemental tables aac.01977-25-s0003.docx Tables S1 to S3.

ASM does not own the copyrights to Supplemental Material that may be linked to, or accessed through, an article. The authors have granted ASM a non-exclusive, world-wide license to publish the Supplemental Material files. Please contact the corresponding author directly for reuse.

## References

[1] Staras SA, Dollard SC, Radford KW, Flanders WD, Pass RF, Cannon MJ. 2006. Seroprevalence of cytomegalovirus infection in the United States, 1988-1994. Clin Infect Dis 43:1143–1151. doi:10.1086/508173 17029132

[2] Demmler GJ. 1991. Infectious diseases society of America and centers for disease control. summary of a workshop on surveillance for congenital cytomegalovirus disease. Rev Infect Dis 13:315–329. doi:10.1093/clinids/13.2.315 1645882

[3] Barbi M, Binda S, Caroppo S, Ambrosetti U, Corbetta C, Sergi P. 2003. A wider role for congenital cytomegalovirus infection in sensorineural hearing loss. Pediatr Infect Dis J 22:39–42. doi:10.1097/00006454-200301000-00012 12544407

[4] Boppana SB, Fowler KB, Britt WJ, Stagno S, Pass RF. 1999. Symptomatic congenital cytomegalovirus infection in infants born to mothers with preexisting immunity to cytomegalovirus. Pediatrics 104:55–60. doi:10.1542/peds.104.1.55 10390260

[5] Yow MD, Demmler GJ. 1992. Congenital cytomegalovirus disease--20 years is long enough. N Engl J Med 326:702–703. doi:10.1056/NEJM199203053261010 1310526

[6] Griffiths PD, Clark DA, Emery VC. 2000. Betaherpesviruses in transplant recipients. J Antimicrob Chemother 45 Suppl T3:29–34. doi:10.1093/jac/45.suppl_4.29 10855769

[7] Kovacs A, Schluchter M, Easley K, Demmler G, Shearer W, Russa PL, Pitt J, Cooper E, Goldfarb J, Hodes D, Kattan M, McIntosh K. 1999. Cytomegalovirus infection and HIV-1 disease progression in infants born to HIV-1–infected women. N Engl J Med 341:77–84. doi:10.1056/NEJM199907083410203 10395631 PMC4280563

[8] Sabin CA, Phillips AN, Lee CA, Janossy G, Emery V, Griffiths PD. 1995. The effect of CMV infection on progression of human immunodeficiency virus disease is a cohort of haemophilic men followed for up to 13 years from seroconversion. Epidemiol Infect 114:361–372. doi:10.1017/s095026880005799x 7705496 PMC2271271

[9] Sabin CA, Devereux HL, Clewley G, Emery VC, Phillips AN, Loveday C, Lee CA, Griffiths PD. 2000. Cytomegalovirus seropositivity and human immunodeficiency virus type 1 RNA levels in individuals with hemophilia. J Infect Dis 181:1800–1803. doi:10.1086/315476 10823788

[10] Khamduang W, Jourdain G, Sirirungsi W, Layangool P, Kanjanavanit S, Krittigamas P, Pagdi K, Somsamai R, Sirinontakan S, Hinjiranandana T, Ardonk W, Hongsiriwon S, Nanta S, Borkird T, Lallemant M, McIntosh K, Ngo-Giang-Huong N, Program for HIV Prevention and Treatment (PHPT) Study Group. 2011. The interrelated transmission of HIV-1 and cytomegalovirus during gestation and delivery in the offspring of HIV-infected mothers. J Acquir Immune Defic Syndr 58:188–192. doi:10.1097/QAI.0b013e31822d0433 21792064 PMC3237680

[11] Jabs DA, Martin BK, Forman MS, Cytomegalovirus Retinitis and Viral Resistance Research Group. 2010. Mortality associated with resistant cytomegalovirus among patients with cytomegalovirus retinitis and AIDS. Ophthalmology 117:128–132. doi:10.1016/j.ophtha.2009.06.016 19818505 PMC2815221

[12] Steininger C. 2007. Novel therapies for cytomegalovirus disease. Recent Pat Antiinfect Drug Discov 2:53–72. doi:10.2174/157489107779561634 18221163

[13] Avery RK, Arav-Boger R, Marr KA, Kraus E, Shoham S, Lees L, Trollinger B, Shah P, Ambinder R, Neofytos D, Ostrander D, Forman M, Valsamakis A. 2016. Outcomes in transplant recipients treated with foscarnet for ganciclovir-resistant or refractory cytomegalovirus infection. Transplantation 100:e74–80. doi:10.1097/TP.0000000000001418 27495775 PMC5030152

[14] Schreiber A, Härter G, Schubert A, Bunjes D, Mertens T, Michel D. 2009. Antiviral treatment of cytomegalovirus infection and resistant strains. Expert Opin Pharmacother 10:191–209. doi:10.1517/14656560802678138 19236193

[15] Chou SW. 2001. Cytomegalovirus drug resistance and clinical implications. Transpl Infect Dis 3 Suppl 2:20–24. doi:10.1034/j.1399-3062.2001.00004.x 11926745

[16] Kimberlin DW, Lin C-Y, Sánchez PJ, Demmler GJ, Dankner W, Shelton M, Jacobs RF, Vaudry W, Pass RF, Kiell JM, Soong S, Whitley RJ, National institute of allergy and infectious diseases collaborative antiviral study group. 2003. Effect of ganciclovir therapy on hearing in symptomatic congenital cytomegalovirus disease involving the central nervous system: a randomized, controlled trial. J Pediatr 143:16–25. doi:10.1016/s0022-3476(03)00192-6 12915819

[17] Kimberlin DW, Jester PM, Sánchez PJ, Ahmed A, Arav-Boger R, Michaels MG, Ashouri N, Englund JA, Estrada B, Jacobs RF, et al.. 2015. Valganciclovir for symptomatic congenital cytomegalovirus disease. N Engl J Med 372:933–943. doi:10.1056/NEJMoa1404599 25738669 PMC4401811

[18] Chou S, Ercolani RJ, Lanier ER. 2016. Novel cytomegalovirus UL54 DNA polymerase gene mutations selected in vitro that confer brincidofovir resistance. Antimicrob Agents Chemother 60:3845–3848. doi:10.1128/AAC.00214-16 27044553 PMC4879386

[19] Chou S. 2015. Rapid in vitro evolution of human cytomegalovirus UL56 mutations that confer letermovir resistance. Antimicrob Agents Chemother 59:6588–6593. doi:10.1128/AAC.01623-15 26259791 PMC4576131

[20] Chemaly RF, Ullmann AJ, Stoelben S, Richard MP, Bornhäuser M, Groth C, Einsele H, Silverman M, Mullane KM, Brown J, Nowak H, Kölling K, Stobernack HP, Lischka P, Zimmermann H, Rübsamen-Schaeff H, Champlin RE, Ehninger G, AIC246 Study Team. 2014. Letermovir for cytomegalovirus prophylaxis in hematopoietic-cell transplantation. N Engl J Med 370:1781–1789. doi:10.1056/NEJMoa1309533 24806159

[21] Marty FM, Ljungman P, Chemaly RF, Maertens J, Dadwal SS, Duarte RF, Haider S, Ullmann AJ, Katayama Y, Brown J, Mullane KM, Boeckh M, Blumberg EA, Einsele H, Snydman DR, Kanda Y, DiNubile MJ, Teal VL, Wan H, Murata Y, Kartsonis NA, Leavitt RY, Badshah C. 2017. Letermovir prophylaxis for cytomegalovirus in hematopoietic-cell transplantation. N Engl J Med 377:2433–2444. doi:10.1056/NEJMoa1706640 29211658

[22] Frietsch JJ, Michel D, Stamminger T, Hunstig F, Birndt S, Schnetzke U, Scholl S, Hochhaus A, Hilgendorf I. 2019. In vivo emergence of UL56 C325Y cytomegalovirus resistance to letermovir in a patient with acute myeloid leukemia after hematopoietic cell transplantation. Mediterr J Hematol Infect Dis 11:e2019001. doi:10.4084/MJHID.2019.001 30671207 PMC6328044

[23] Winston DJ, Saliba F, Blumberg E, Abouljoud M, Garcia-Diaz JB, Goss JA, Clough L, Avery R, Limaye AP, Ericzon BG, Navasa M, Troisi RI, Chen H, Villano SA, Uknis ME, 1263-301 Clinical Study Group. 2012. Efficacy and safety of maribavir dosed at 100 mg orally twice daily for the prevention of cytomegalovirus disease in liver transplant recipients: a randomized, double-blind, multicenter controlled trial. Am J Transplant 12:3021–3030. doi:10.1111/j.1600-6143.2012.04231.x 22947426

[24] Winston DJ, Young J-AH, Pullarkat V, Papanicolaou GA, Vij R, Vance E, Alangaden GJ, Chemaly RF, Petersen F, Chao N, Klein J, Sprague K, Villano SA, Boeckh M. 2008. Maribavir prophylaxis for prevention of cytomegalovirus infection in allogeneic stem cell transplant recipients: a multicenter, randomized, double-blind, placebo-controlled, dose-ranging study. Blood 111:5403–5410. doi:10.1182/blood-2007-11-121558 18285548 PMC5726327

[25] Papanicolaou GA, Silveira FP, Langston AA, Pereira MR, Avery RK, Uknis M, Wijatyk A, Wu J, Boeckh M, Marty FM, Villano S. 2019. Maribavir for refractory or resistant cytomegalovirus infections in hematopoietic-cell or solid-organ transplant recipients: a randomized, dose-ranging, double-blind, phase 2 study. Clin Infect Dis 68:1255–1264. doi:10.1093/cid/ciy706 30329038 PMC6451997

[26] Avery RK, Alain S, Alexander BD, Blumberg EA, Chemaly RF, Cordonnier C, Duarte RF, Florescu DF, Kamar N, Kumar D, Maertens J, Marty FM, Papanicolaou GA, Silveira FP, Witzke O, Wu J, Sundberg AK, Fournier M, Solstice trial investigators. 2022. Maribavir for refractory cytomegalovirus infections with or without resistance post-transplant: results from a phase 3 randomized clinical trial. Clin Infect Dis 75:690–701. doi:10.1093/cid/ciab988 34864943 PMC9464078

[27] Blumberg EA, Witzke O, Harber M, Ison MG, Saliba F, Kamar N, Sundberg AK, Gu J, Kumar D, La Hoz RM. 2025. Maribavir for refractory cytomegalovirus infection (with or without resistance) in solid organ transplant recipients: subgroup analysis of the phase 3 randomized solstice study. J Heart Lung Transplant 44:986–994. doi:10.1016/j.healun.2024.11.026 39613120

[28] Chou S. 1999. Antiviral drug resistance in human cytomegalovirus. Transpl Infect Dis 1:105–114. doi:10.1034/j.1399-3062.1999.010204.x 11428978

[29] Kapoor A, Ghosh AK, Forman M, Hu X, Ye W, Southall N, Marugan J, Keyes RF, Smith BC, Meyers DJ, Ferrer M, Arav-Boger R. 2020. Validation and characterization of five distinct novel inhibitors of human cytomegalovirus. J Med Chem 63:3896–3907. doi:10.1021/acs.jmedchem.9b01501 32191456 PMC7386824

[30] He R, Sandford G, Hayward GS, Burns WH, Posner GH, Forman M, Arav-Boger R. 2011. Recombinant luciferase-expressing human cytomegalovirus (CMV) for evaluation of CMV inhibitors. Virol J 8:40. doi:10.1186/1743-422X-8-40 21269468 PMC3041771

[31] Forman MS, Vaidya D, Bolorunduro O, Diener-West M, Pass RF, Arav-Boger R. 2017. Cytomegalovirus kinetics following primary infection in healthy women. J Infect Dis 215:1523–1526. doi:10.1093/infdis/jix188 28431127 PMC5461424

[32] Schleiss MR, Bourne N, Bravo FJ, Jensen NJ, Bernstein DI. 2003. Quantitative-competitive PCR monitoring of viral load following experimental guinea pig cytomegalovirus infection. J Virol Methods 108:103–110. doi:10.1016/s0166-0934(02)00265-3 12565160

[33] James SH, Price NB, Hartline CB, Lanier ER, Prichard MN. 2013. Selection and recombinant phenotyping of a novel CMX001 and cidofovir resistance mutation in human cytomegalovirus. Antimicrob Agents Chemother 57:3321–3325. doi:10.1128/AAC.00062-13 23650158 PMC3697342

[34] Goldner T, Hewlett G, Ettischer N, Ruebsamen-Schaeff H, Zimmermann H, Lischka P. 2011. The novel anticytomegalovirus compound AIC246 (Letermovir) inhibits human cytomegalovirus replication through a specific antiviral mechanism that involves the viral terminase. J Virol 85:10884–10893. doi:10.1128/JVI.05265-11 21752907 PMC3187482

[35] Tischer BK, von Einem J, Kaufer B, Osterrieder N. 2006. Two-step red-mediated recombination for versatile high-efficiency markerless DNA manipulation in Escherichia coli. Biotechniques 40:191–197. doi:10.2144/000112096 16526409

[36] Borst EM, Hahn G, Koszinowski UH, Messerle M. 1999. Cloning of the human cytomegalovirus (HCMV) genome as an infectious bacterial artificial chromosome in Escherichia coli: a new approach for construction of HCMV mutants. J Virol 73:8320–8329. doi:10.1128/JVI.73.10.8320-8329.1999 10482582 PMC112849

[37] Chou S. 2008. Cytomegalovirus UL97 mutations in the era of ganciclovir and maribavir. Rev Med Virol 18:233–246. doi:10.1002/rmv.574 18383425

[38] Chou S, Bowlin TL. 2011. Cytomegalovirus UL97 mutations affecting cyclopropavir and ganciclovir susceptibility. Antimicrob Agents Chemother 55:382–384. doi:10.1128/AAC.01259-10 21041510 PMC3019618

[39] Eizuru Y. 2003. Development of new antivirals for herpesviruses. Antivir Chem Chemother 14:299–308. doi:10.1177/095632020301400602 14968936

[40] Cushing TD, Adrian J, Chen X, DiMaio H, Doughan B, Flygare J, Liang L, Mayorga V, Miao S, Mellon H, Peterson MG, Powers JP, Spector F, Stein C, Wright M, Xu D, Ye Q, Jaen J. 2006. Discovery of a novel series of inhibitors of human cytomegalovirus primase. Bioorg Med Chem Lett 16:4879–4883. doi:10.1016/j.bmcl.2006.06.066 16814545

[41] Baranovskiy AG, He Q, Suwa Y, Morstadt LM, Babayeva ND, Lim CJ, Tahirov TH. 2025. Structural basis of herpesvirus helicase-primase inhibition by pritelivir and amenamevir. Sci Adv 11:eadz1989. doi:10.1126/sciadv.adz1989 41202142 PMC12594203

[42] Beyer DC, Ghoneim MK, Spies M. 2013. Structure and mechanisms of SF2 DNA helicases. Adv Exp Med Biol 767:47–73. doi:10.1007/978-1-4614-5037-5_3 23161006

[43] Weller SK, Kuchta RD. 2013. The DNA helicase-primase complex as a target for herpes viral infection. Expert Opin Ther Targets 17:1119–1132. doi:10.1517/14728222.2013.827663 23930666 PMC4098783

[44] Spector FC, Liang L, Giordano H, Sivaraja M, Peterson MG. 1998. Inhibition of herpes simplex virus replication by a 2-amino thiazole via interactions with the helicase component of the UL5-UL8-UL52 complex. J Virol 72:6979–6987. doi:10.1128/JVI.72.9.6979-6987.1998 9696789 PMC109917

[45] Crute JJ, Grygon CA, Hargrave KD, Simoneau B, Faucher A-M, Bolger G, Kibler P, Liuzzi M, Cordingley MG. 2002. Herpes simplex virus helicase-primase inhibitors are active in animal models of human disease. Nat Med 8:386–391. doi:10.1038/nm0402-386 11927945

[46] Duan J, Liuzzi M, Paris W, Liard F, Browne A, Dansereau N, Simoneau B, Faucher A-M, Cordingley MG. 2003. Oral bioavailability and in vivo efficacy of the helicase-primase inhibitor BILS 45 BS against acyclovir-resistant herpes simplex virus type 1. Antimicrob Agents Chemother 47:1798–1804. doi:10.1128/AAC.47.6.1798-1804.2003 12760851 PMC155846

[47] Kleymann G, Fischer R, Betz UAK, Hendrix M, Bender W, Schneider U, Handke G, Eckenberg P, Hewlett G, Pevzner V, Baumeister J, Weber O, Henninger K, Keldenich J, Jensen A, Kolb J, Bach U, Popp A, Mäben J, Frappa I, Haebich D, Lockhoff O, Rübsamen-Waigmann H. 2002. New helicase-primase inhibitors as drug candidates for the treatment of herpes simplex disease. Nat Med 8:392–398. doi:10.1038/nm0402-392 11927946

[48] Chono K, Katsumata K, Kontani T, Kobayashi M, Sudo K, Yokota T, Konno K, Shimizu Y, Suzuki H. 2010. ASP2151, a novel helicase-primase inhibitor, possesses antiviral activity against varicella-zoster virus and herpes simplex virus types 1 and 2. J Antimicrob Chemother 65:1733–1741. doi:10.1093/jac/dkq198 20534624

[49] Shiraki K, Yasumoto S, Toyama N, Fukuda H. 2021. Amenamevir, a helicase-primase inhibitor, for the optimal treatment of herpes zoster. Viruses 13:1547. doi:10.3390/v13081547 34452412 PMC8402822

[50] Sasaki S-I, Miyazaki D, Haruki T, Yamamoto Y, Kandori M, Yakura K, Suzuki H, Inoue Y. 2013. Efficacy of herpes virus helicase-primase inhibitor, ASP2151, for treating herpes simplex keratitis in mouse model. Br J Ophthalmol 97:498–503. doi:10.1136/bjophthalmol-2012-302062 23361434

[51] Bernstein DI, Sawtell NM, Bravo FJ, Dixon DA, Gege C, Kleymann G. 2023. Intermittent therapy with helicase-primase inhibitor IM-250 efficiently controls recurrent herpes disease and reduces reactivation of latent HSV. Antiviral Res 219:105733. doi:10.1016/j.antiviral.2023.105733 37858763

[52] Chee MS, Bankier AT, Beck S, Bohni R, Brown CM, Cerny R, Horsnell T, Hutchison CA 3rd, Kouzarides T, Martignetti JA. 1990. Analysis of the protein-coding content of the sequence of human cytomegalovirus strain AD169. Curr Top Microbiol Immunol 154:125–169. doi:10.1007/978-3-642-74980-3_6 2161319

[53] Smith JA, Jairath S, Crute JJ, Pari GS. 1996. Characterization of the human cytomegalovirus UL105 gene and Identification of the putative helicase protein. Virology 220:251–255. doi:10.1006/viro.1996.0310 8659123

[54] Smith JA, Pari GS. 1995. Human cytomegalovirus UL102 gene. J Virol 69:1734–1740. doi:10.1128/jvi.69.3.1734-1740.1995 7853511 PMC188777

[55] McMahon TP, Anders DG. 2002. Interactions between human cytomegalovirus helicase-primase proteins. Virus Res 86:39–52. doi:10.1016/s0168-1702(02)00054-0 12076828

[56] Ligat G, Da Re S, Alain S, Hantz S. 2018. Identification of amino acids essential for viral replication in the HCMV Helicase-primase complex. Front Microbiol 9:2483. doi:10.3389/fmicb.2018.02483 30405556 PMC6205958

[57] Schleiss MR, McVoy MA. 2010. Guinea pig cytomegalovirus (GPCMV): a model for the study of the prevention and treatment of maternal-fetal transmission. Future Virol 5:207–217. doi:10.2217/fvl.10.8 23308078 PMC3539792

[58] Sukla S, Biswas S, Birkmann A, Lischka P, Zimmermann H, Field HJ. 2010. Mismatch primer-based PCR reveals that helicase-primase inhibitor resistance mutations pre-exist in herpes simplex virus type 1 clinical isolates and are not induced during incubation with the inhibitor. J Antimicrob Chemother 65:1347–1352. doi:10.1093/jac/dkq135 20453068 PMC2835512

