# Supplementary material for: A novel inhibitor of human cytomegalovirus acts through a specific antiviral mechanism that involves the helicase-primase complex
Source: Antimicrob Agents Chemother. 2026 Jul 13;70(8):e01977-25. doi: 10.1128/aac.01977-25 (PMC13435774; doi:10.1128/aac.01977-25)
Supplement: Supplementary file 1 [file aac.01977-25-s0001.pdf]

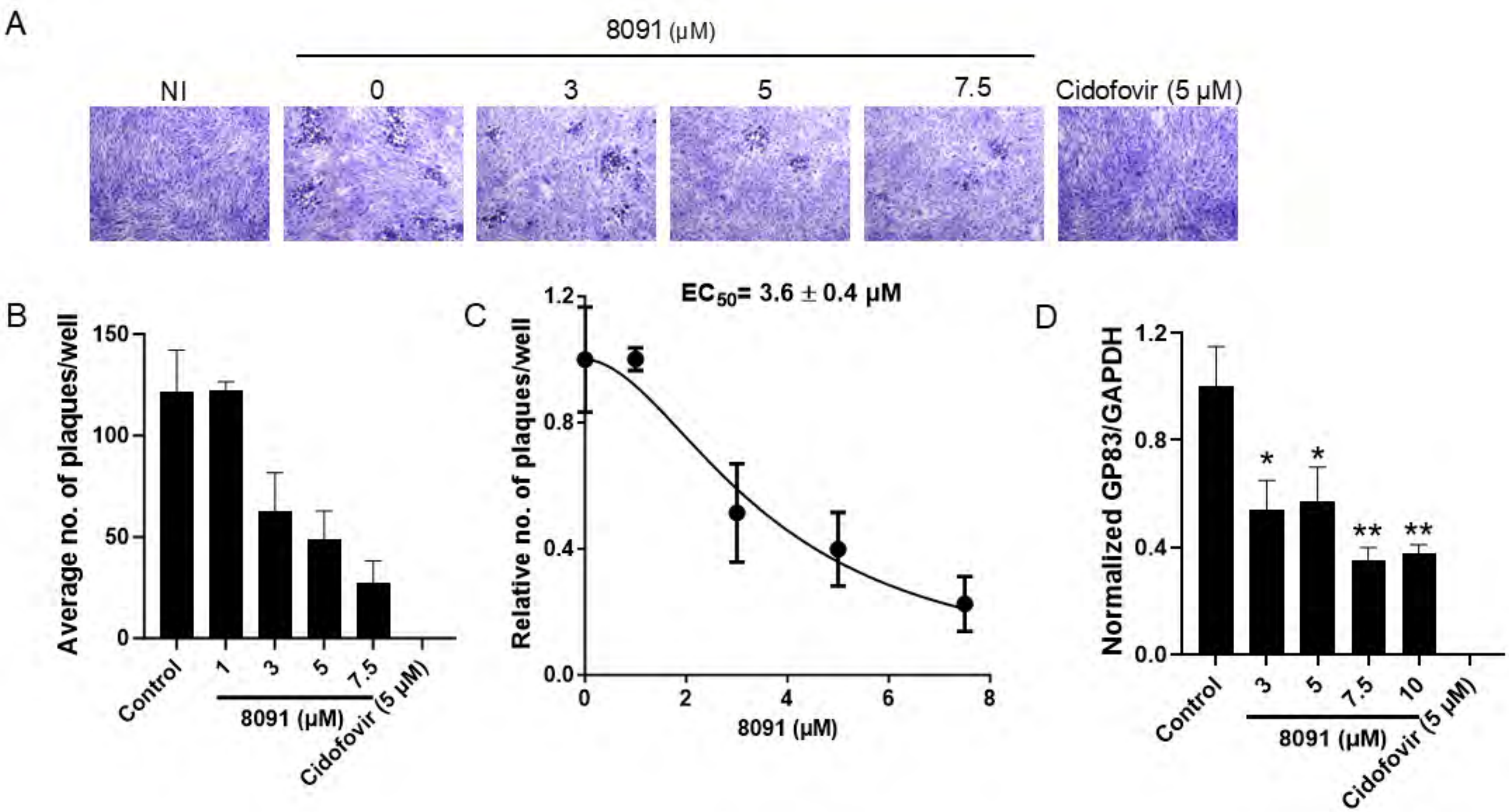
A
8091 (μM)
NI
0
3
5
7.5
Cidofovir (5 μM)
B
Average no. of plaques/well
150
100
50
0
Control
1
3
5
7.5
8091 (μM)
Cidofovir (5 μM)
C
EC50= 3.6 ± 0.4 μM
Relative no. of plaques/well
1.2
0.8
0.4
0.0
0
2
4
6
8
8091 (μM)
D
Normalized GP83/GAPDH
1.2
0.8
0.4
0.0
*
*
**
**
Control
3
5
7.5
10
8091 (μM)
Cidofovir (5 μM)

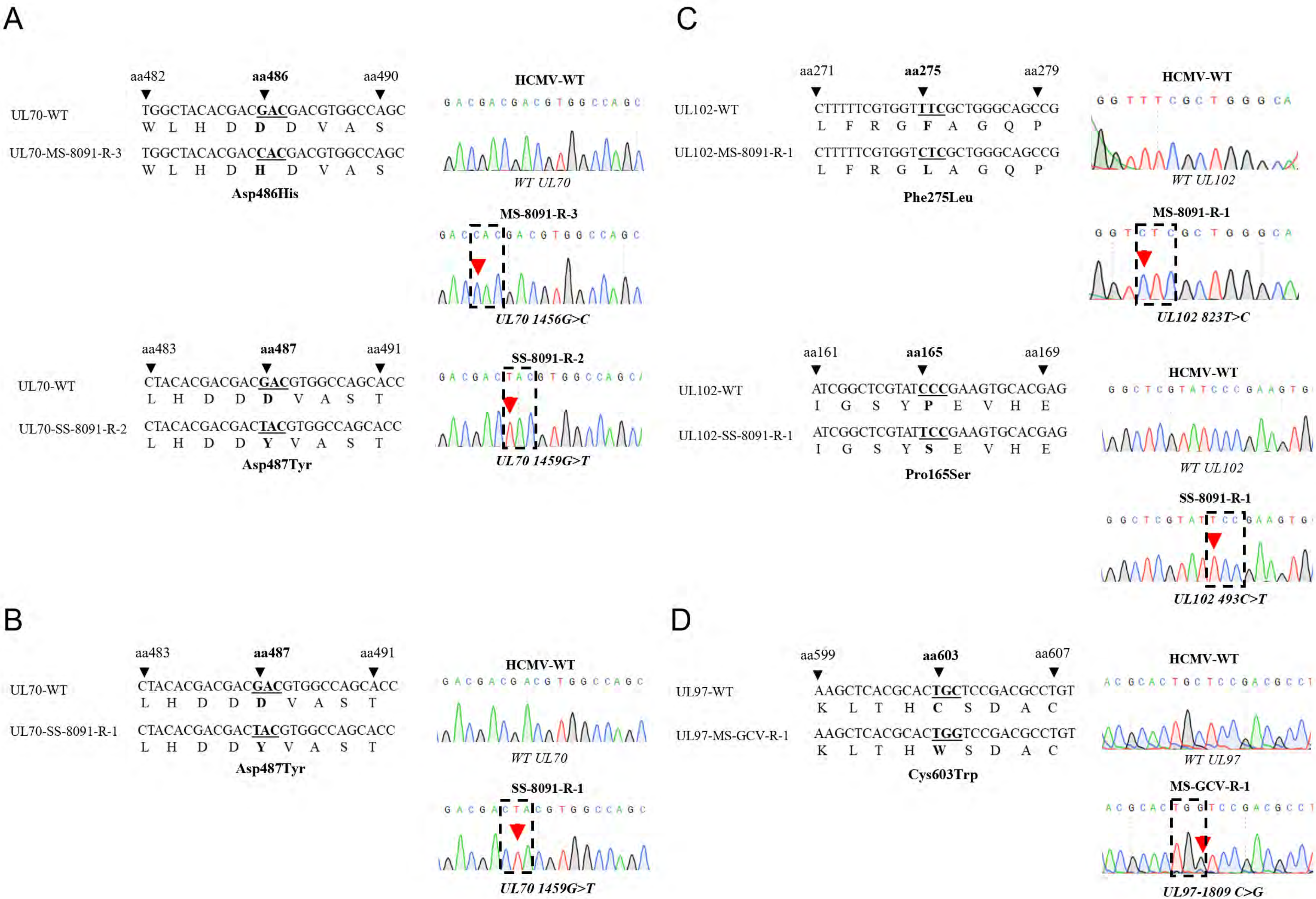

A
aa482
aa486
aa490
UL70-WT
TGGCTACACGACGACGACGTGGCCAGC
W L H D D D V A S
UL70-MS-8091-R-3
TGGCTACACGACCACGACGTGGCCAGC
W L H D H D V A S
Asp486His
HCMV-WT
WT UL70
MS-8091-R-3
UL70 1456G>C
aa483
aa487
aa491
UL70-WT
CTACACGACGACGACGTGGCCAGCACC
L H D D D V A S T
UL70-SS-8091-R-2
CTACACGACGACTACGTGGCCAGCACC
L H D D Y V A S T
Asp487Tyr
SS-8091-R-2
UL70 1459G>T
B
aa483
aa487
aa491
UL70-WT
CTACACGACGACGACGTGGCCAGCACC
L H D D D V A S T
UL70-SS-8091-R-1
CTACACGACGACTACGTGGCCAGCACC
L H D D Y V A S T
Asp487Tyr
HCMV-WT
WT UL70
SS-8091-R-1
UL70 1459G>T
C
aa271
aa275
aa279
UL102-WT
CTTTTTCGTGGTTTCGCTGGGCAGCCG
L F R G F A G Q P
UL102-MS-8091-R-1
CTTTTTCGTGGTCTCGCTGGGCAGCCG
L F R G L A G Q P
Phe275Leu
HCMV-WT
WT UL102
MS-8091-R-1
UL102 823T>C
aa161
aa165
aa169
UL102-WT
ATCGGCTCGTATCCCGAAGTGCACGAG
I G S Y P E V H E
UL102-SS-8091-R-1
ATCGGCTCGTATTCCGAAGTGCACGAG
I G S Y S E V H E
Pro165Ser
HCMV-WT
WT UL102
SS-8091-R-1
UL102 493C>T
D
aa599
aa603
aa607
UL97-WT
AAGCTCACGCACTGCTCCGACGCCTGT
K L T H C S D A C
UL97-MS-GCV-R-1
AAGCTCACGCACTGGTCCGACGCCTGT
K L T H W S D A C
Cys603Trp
HCMV-WT
WT UL97
MS-GCV-R-1
UL97-1809 C>G

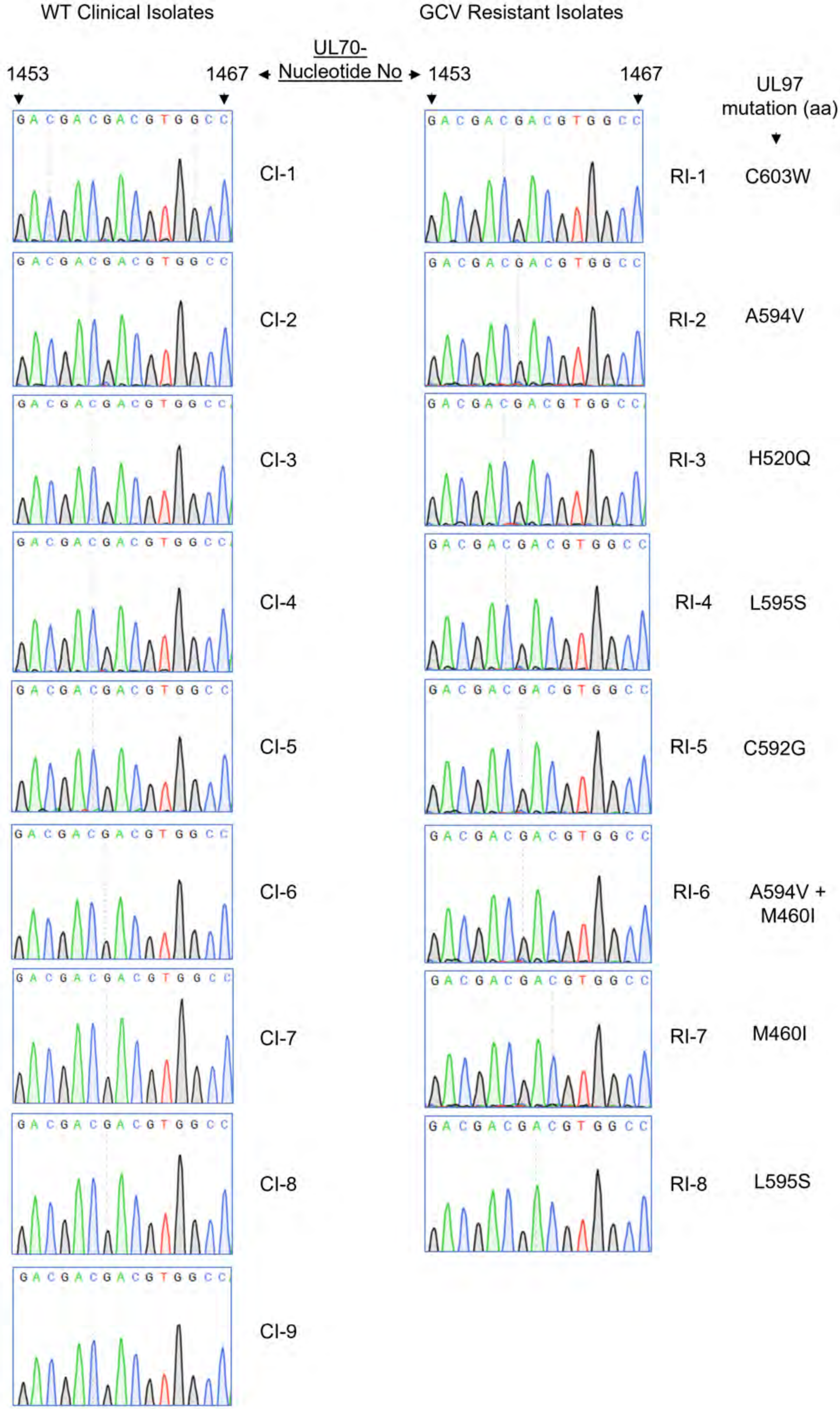
WT Clinical Isolates
GCV Resistant Isolates
UL70-
Nucleotide No
1453
1467
UL97
mutation (aa)
GACGACGACGTGGCC
CI-1
CI-2
CI-3
CI-4
CI-5
CI-6
CI-7
CI-8
CI-9
RI-1 C603W
RI-2 A594V
RI-3 H520Q
RI-4 L595S
RI-5 C592G
RI-6 A594V + M460I
RI-7 M460I
RI-8 L595S

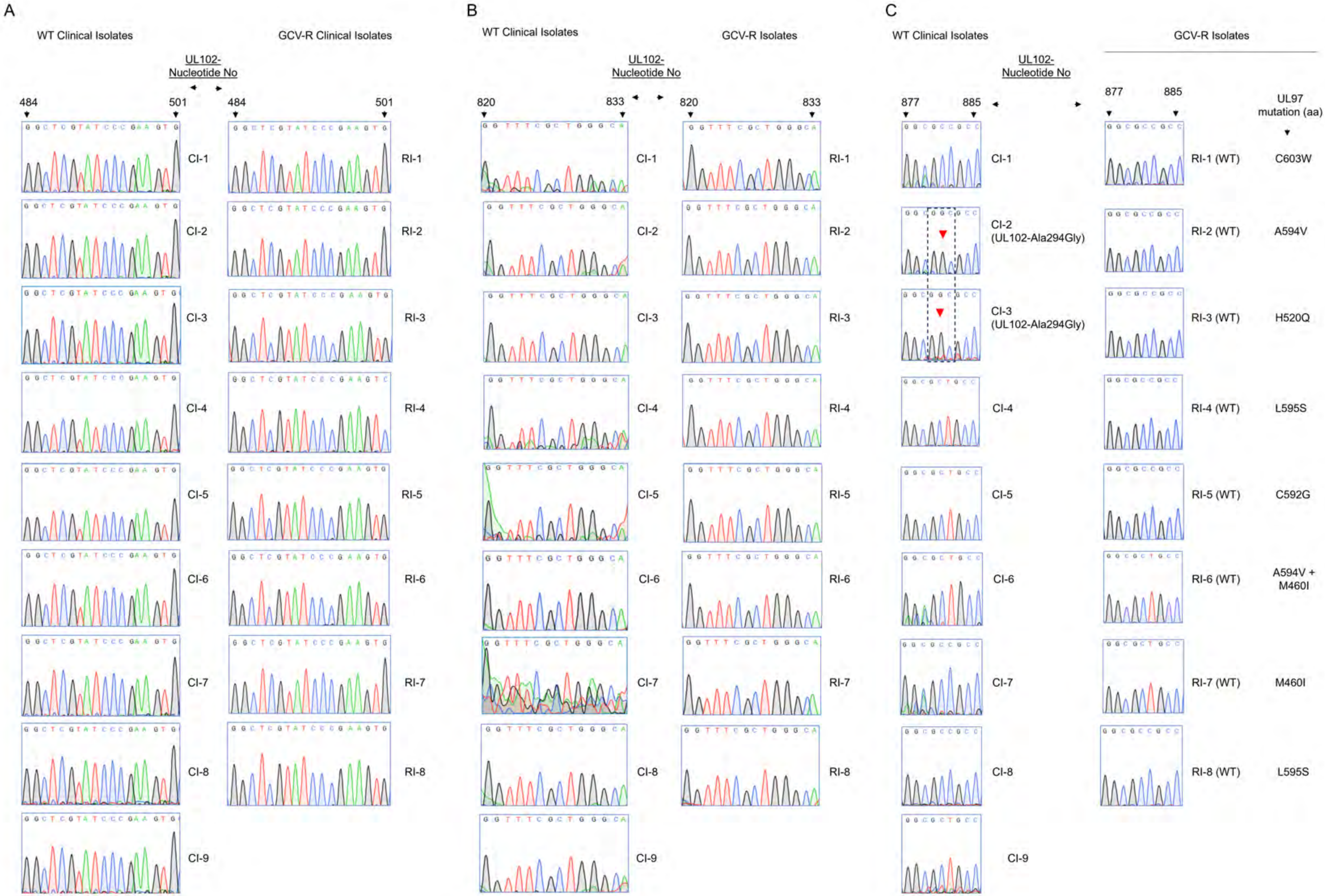
A
WT Clinical Isolates
GCV-R Clinical Isolates
UL102-
Nucleotide No
484
501
484
501
CI-1
CI-2
CI-3
CI-4
CI-5
CI-6
CI-7
CI-8
CI-9
RI-1
RI-2
RI-3
RI-4
RI-5
RI-6
RI-7
RI-8
B
WT Clinical Isolates
GCV-R Isolates
UL102-
Nucleotide No
820
833
820
833
CI-1
CI-2
CI-3
CI-4
CI-5
CI-6
CI-7
CI-8
CI-9
RI-1
RI-2
RI-3
RI-4
RI-5
RI-6
RI-7
RI-8
C
WT Clinical Isolates
GCV-R Isolates
UL102-
Nucleotide No
877
885
877
885
UL97
mutation (aa)
CI-1
CI-2
(UL102-Ala294Gly)
CI-3
(UL102-Ala294Gly)
CI-4
CI-5
CI-6
CI-7
CI-8
CI-9
RI-1 (WT)
RI-2 (WT)
RI-3 (WT)
RI-4 (WT)
RI-5 (WT)
RI-6 (WT)
RI-7 (WT)
RI-8 (WT)
C603W
A594V
H520Q
L595S
C592G
A594V +
M460I
M460I
L595S

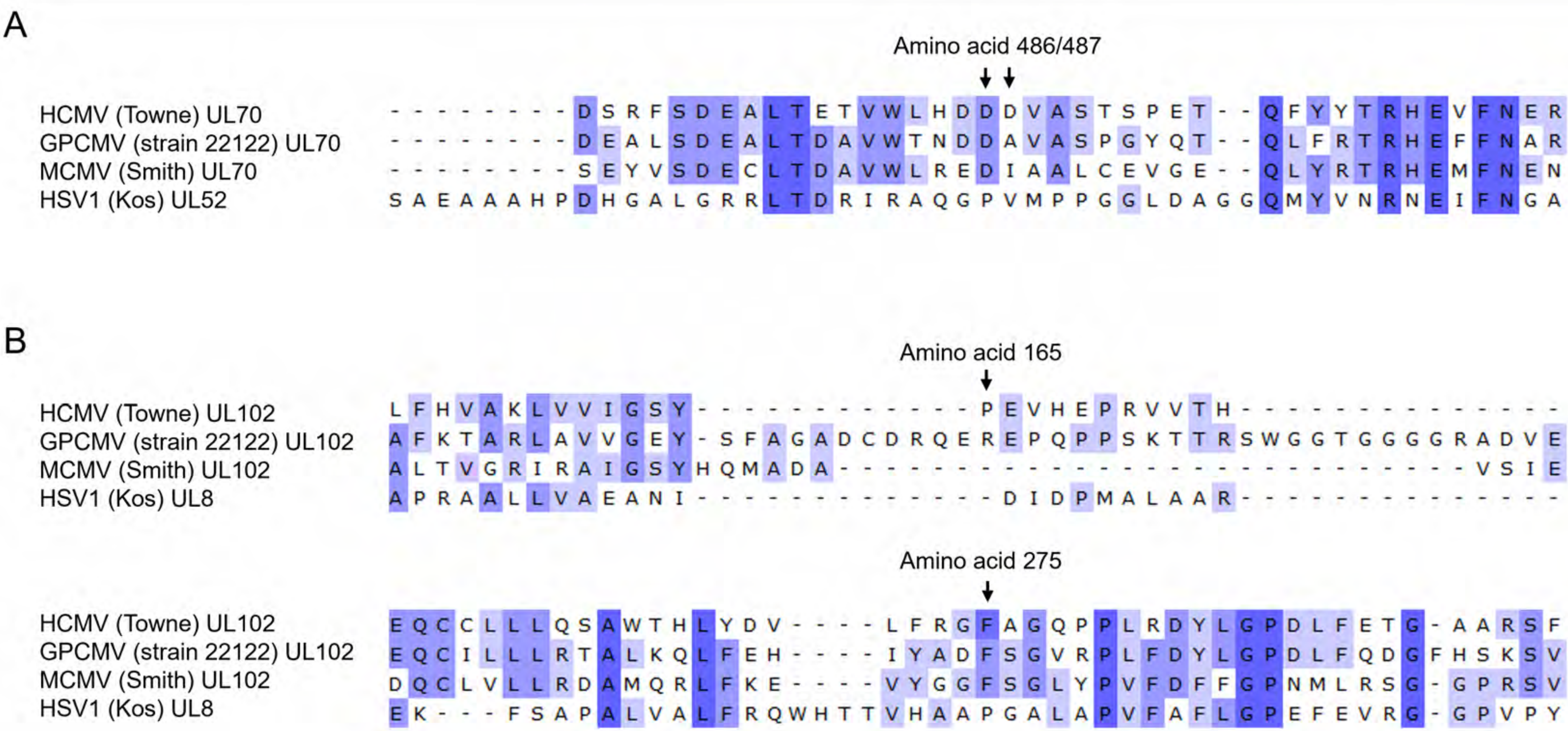
A
Amino acid 486/487
HCMV (Towne) UL70 --------DSRFSDEALTETVWLHDDDVASTSPET--QFYYTRHEVFNER
GPCMV (strain 22122) UL70 --------DEALSDEALTDAVWTNDDAVASPGYQT--QLFRTRHEFFNAR
MCMV (Smith) UL70 --------SEYVSDECLTDAVWLREDIAALCEVGE--QLYRTRHEMFNEN
HSV1 (Kos) UL52 SAEAAAHPDHGALGRRLTDRIRAQGPVMPPGGLDAGGQMYVNRNEIFNGA
B
Amino acid 165
HCMV (Towne) UL102 LFHVAKLVVIGSY-------------PEVHEPRVVTH-------------
GPCMV (strain 22122) UL102 AFKTARLAVVGEY-SFAGADCDRQEREPQPPSKTTRSWGGTGGGGRADVE
MCMV (Smith) UL102 ALTVGRIRAIGSYHQMADA---------------------------VSIE
HSV1 (Kos) UL8 APRAALLVAEANI-------------DIDPMALAAR--------------
Amino acid 275
HCMV (Towne) UL102 EQCCLLLQSAWTHLYDV----LFRGFAGQPPLRDYLGPDLFETG-AARSF
GPCMV (strain 22122) UL102 EQCILLLRTALKQLFEH----IYADFSGVRPLFDYLGPDLFQDGFHSKSV
MCMV (Smith) UL102 DQCLVLLRDAMQRLFKE----VYGGFSGLYPVFDFFGPNMLRSG-GPRSV
HSV1 (Kos) UL8 EK---FSAPALVALFRQWHTTVHAAPGALAPVFAFLGPEFEVRG-GPVPY
