# Supplementary material for: A novel inhibitor of human cytomegalovirus acts through a specific antiviral mechanism that involves the helicase-primase complex
Source: Antimicrob Agents Chemother. 2026 Jul 13;70(8):e01977-25. doi: 10.1128/aac.01977-25 (PMC13435774; doi:10.1128/aac.01977-25)
Supplement: Supplementary file 2 [file aac.01977-25-s0002.docx]

**Supplemental figures:**

**Supplemental Figure 1: Activity of MLS8091 against GPCMV**. **A)** A plaque assay was performed with the indicated concentrations of MLS8091. The average and relative numbers of plaques were plotted **(B, C)**. **D)** Quantitative GP83 PCR was performed with the indicated concentrations of MLS8091. Cidofovir was used as a positive control. Statistical significance was compared to the infected control.

**Supplemental Figure 2**: **Sequence alignments and chromatograms of UL70, WT, and mutants (A, B), UL102, WT, and mutants (C), and UL97 WT and mutant (D).**

**Supplemental Figure 3: Sequence analysis of UL70 in the region of the identified mutations.** Left: WT clinical isolates lacking known UL97 mutations; right: GCV-resistant clinical isolates with known UL97 mutations.

**Supplemental Figure 4: Sequence analysis of UL102 in the region of the identified mutations. A, B)** The areas of UL102 harboring mutations induced by MLS-8091 were sequenced from WT clinical isolates (no UL97 mutations) and GCV-resistant clinical isolates (known UL97 mutations). **C**) A mutation (Ala 294Gly) in UL102 was identified in two clinical isolates (Cl2 and Cl3). The UL97 amino acid (aa) mutations associated with GCV resistance are listed on the right.

**Supplemental Figure 5: Low sequence homology in HCMV, GPCMV, MCMV, and HSV-1 in MLS8091-selected mutations**. Amino acid alignment of UL70 **(A)** and UL102 **(B)** HCMV Towne, GPCMV, MCMV, and HSV-1 sequences. Arrows indicate the MLS8091-resistant mutations in HCMV Towne. The percent homology of the full UL70 sequences among HCMV, GPCMV, MCMV, and HSV-1 was 45.2%, 41.3%, and 28.7%, respectively, as calculated using ExPASy. The percent homology of full-length UL102 was 33.2%, 29.4%, and 28.6%.
