# Supplementary material for: A novel inhibitor of human cytomegalovirus acts through a specific antiviral mechanism that involves the helicase-primase complex
Source: Antimicrob Agents Chemother. 2026 Jul 13;70(8):e01977-25. doi: 10.1128/aac.01977-25 (PMC13435774; doi:10.1128/aac.01977-25)
Supplement: Supplementary file 3 [file aac.01977-25-s0003.docx]

**Supplemental Table 1: Primer sequences used for introducing point mutations in AD169 BAC**

| **Primer name** | **Sequence** |  |
| --- | --- | --- |
| UL70 1459 G>T FP | 5'-CGAGGCCCTGACCGAGACCGTGTGGCTACACGACGACTACGT GGCCAGCACCAGCCCCGAAGGATGACGACGATAAGTAGGG-3' | |
| UL70 1459 G>T RP | 5'-GCGTGTAGTAGAACTGTGTCTCGGGGCTGGTGCTGGCCACGTAG TCGTCGTGTAGCCACACAACCAATTAACCAATTCTGATTAG-3' | |
| UL70 1456 G>C-FP | 5'-CGAGGCCCTGACCGAGACCGTGTGGCTACACGACCACGACGTG GCCAGCACCAGCCCCGAAGGATGACGACGATAAGTAGGG-3' | |
| UL70 1456 G>C-RP | 5'-GCGTGTAGTAGAACTGTGTCTCGGGGCTGGTGCTGGCCACGT CGTGGTCGTGTAGCCACACAACCAATTAACCAATTCTGATTAG-3' | |
| UL102-AD169 493 C>T FP | 5'-TTTCCACGTAGCCAAGCTAGTGGTCATCGGCTCGTATTCCGAA GTGCACGAGCCGCGTGTAGGATGACGACGATAAGTAGGG-3' | |
| UL102-AD169 493 C>T RP | 5'-GTTCCGCGGTATGCGTGACCACACGCGGCTCGTGCACTTCGG AATACGAGCCGATGACCACAACCAATTAACCAATTCTGATTAG-3' | |
| UL102-AD169 823T>C FP | 5'-GGCCTGGACTCACCTTTACGACGTGCTTTTCCGTGGTCTCGCTGG GCAGCCGCCGCTACGAGGATGACGACGATAAGTAGGG-3' | |
| UL102-AD169 823T>C RP | 5'-GGTCCGGCCCCAGGTAGTCGCGTAGCGGCGGCTGCCCAGCGAG ACCACGGAAAAGCACGTCAACCAATTAACCAATTCTGATTAG-3' | |
| *Sequences that anneal with the pEPKan-S plasmid are underlined  FP- forward primer, RP- reverse primer | |  |

**Supplemental Table 2: Primers used for sequencing specific point mutations in AD169, Towne, and in clinical isolates**

| **Primer name** | **Sequence** | **Point mutations tested** |
| --- | --- | --- |
| UL70 Sequencing FP | 5’- GTCTACCGCGTACACCTGC-3’ | UL70 1459 G>T and UL701456 G>C |
| UL70 Sequencing RP | 5’- AGACGCGTCGTAGTCCGC -3’ | UL70 1459 G>T and UL701456 G>C |
| UL102 Sequencing FP | 5’-GGTGCAGATTCAGACGGAC-3’ | UL102 493 C>T |
| UL102 Sequencing RP | 5’- GCACATACTTGTGAGTGCCG -3’ | UL102 493 C>T |
| UL102 Sequencing FP | 5’-TGACCGATGAGCAGTGTTGT-3’ | UL102 823 T>C |
| UL102 Sequencing RP | 5’-GTTCCACCTCCAGCTTGC-3’ | UL102 823 T>C |
| UL102 Sequencing FP | 5'-CTTTGAGGTGCGCGAGGTGT-3' | UL102 823 T>C |
| UL102 Sequencing RP | 5'-CACCACGAGAGCACCTCAG-3' | UL102 823 T>C |

FP- forward primer, RP- reverse primer

**Supplemental Table 3: NGS quality metrics**

| **Sample Detail** | **Total Reads** | **Mapped Reads** | **Mean Depth (genome)** | **Depth at UL70 486–487** | **Depth at UL102 165/275** | **Other Variants Called (outside UL70, UL97, UL102)** |
| --- | --- | --- | --- | --- | --- | --- |
| WT CMV | 3,501,745 | 3,060,177 | 2018.46 | 0 | 0 | 22 |
| GCV- R | 5,359,222 | 1,373,376 | 899.062 | 0 | 0 | 50 |
| MLS8091 – MS (R1) | 4,147,391 | 3,798,672 | 2497.82 | 0 | 1865 | 23 |
| MLS8091 – SS (R1) | 6,817,172 | 4,923,833 | 3238.93 | 2572 | 2564 | 25 |

Bowtie2/2.2.5 was used to align the reads to the human herpesvirus 5 strain Towne reference. Samtools/1.5.0 was then used to convert, sort, and index the alignment file. The following parameters were used *-d 10000* to limit read depth at a position*, -Q 30* to filter out reads with low mapping quality of less than a Phred score of 30, *-uv* to generate an uncompressed output file and *-t DP4* to include the DP4 tag in the VCF info field. This provides the number of forward reference bases, reverse reference bases, forward alternate bases, and reverse alternate bases. Bcftools/1.5 view was used to convert the pileup file to a vcf file, and bcftools/1.5 call was used to generate the genomic variants. The parameters *-m--variants-only* were used to call the alternative model for multiallelic and rare-variant calling, and to print only the variant sites.
